# Nocturnal plant respiration is under strong non-temperature control

**DOI:** 10.1038/s41467-022-33370-1

**Published:** 2022-09-26

**Authors:** Dan Bruhn, Freya Newman, Mathilda Hancock, Peter Povlsen, Martijn Slot, Stephen Sitch, John Drake, Graham P. Weedon, Douglas B. Clark, Majken Pagter, Richard J. Ellis, Mark G. Tjoelker, Kelly M. Andersen, Zorayda Restrepo Correa, Patrick C. McGuire, Lina M. Mercado

**Affiliations:** 1 Department of Chemistry and Bioscience, Aalborg University, Aalborg, Denmark; 2 Faculty of Environment, Science and Economy’, University of Exeter, Exeter, United Kingdom; 3 Smithsonian Tropical Research Institute, Balboa Ancon, Republic of Panama; 4 Department of Sustainable Resources Management, SUNY College of Environmental Science and Forestry, Syracuse, USA; 5 Met Office, Wallingford, United Kingdom; 6 UK Centre for Ecology & Hydrology, Wallingford, United Kingdom; 7 Hawkesbury Institute for the Environment, Western Sydney University, Penrith, Australia; 8 Nanyang Technological Institute, Singapore, Singapore; 9 Grupo Servicios ecosistemicos y cambio climático (SECC), Corporación COL-TREE, Medellin, Colombia; 10 University of Reading, Department of Meteorology and National Centre for Atmospheric Science, Reading, United Kingdom

**Keywords:** Plant physiology, Carbon cycle, Ecophysiology

## Abstract

Most biological rates depend on the rate of respiration. Temperature variation is typically considered the main driver of daily plant respiration rates, assuming a constant daily respiration rate at a set temperature. Here, we show empirical data from 31 species from temperate and tropical biomes to demonstrate that the rate of plant respiration at a constant temperature decreases monotonically with time through the night, on average by 25% after 8 h of darkness. Temperature controls less than half of the total nocturnal variation in respiration. A new universal formulation is developed to model and understand nocturnal plant respiration, combining the nocturnal decrease in the rate of plant respiration at constant temperature with the decrease in plant respiration according to the temperature sensitivity. Application of the new formulation shows a global reduction of 4.5 −6 % in plant respiration and an increase of 7-10% in net primary production for the present-day.

## Abstract (web summary)

Plant respiration at night is assumed to be temperature-controlled. Here, the authors show that temperature controls less than half of the variation in leaf respiration rate at night, and demonstrate how to account for such nocturnal variation in biosphere models.

## Introduction

Respiration is a multi-enzymatic process that is considered the most fundamental biological process^1^ as it underlies other metabolic processes by providing the necessary energy and carbon skeletons. The by-product is CO_2_, which represents a reduction in plant carbon-gain^2^ and at the ecosystem level, it represents high rates of CO_2_ release to the atmosphere^3^. Annually, roughly 30 Gt carbon is emitted to the atmosphere through leaf respiration^4,5^.

A constant rate of respiration (*R*, typically measured in plant studies as CO_2_-efflux per second and in eddy-covariance studies as CO_2_-efflux per 30 min) at a constant temperature (T_o_) underlies numerous models of leaf physiology^6^, scaling of ecosystem components of autotrophic *R* (i.e. leaf, stem and root) for estimation of the net- and gross primary production using biometric methods and eddy covariance^6–11^, estimates of ecosystem *R* and gap filling in eddy covariance studies^12–16^, and Terrestrial Biosphere Models (TBM) of CO_2_ exchange between the Earth and atmosphere^3,17^. In all these disciplines, it is essential to estimate integrals of *R* over any time interval. Models of leaf *R* provided by plant the plant eco-physiological community for ecosystem and global vegetation modelling are mainly driven by temperature (T)^18–21^ due to the assumption of a constant rate of *R* at T_o_ (*R*_To_) throughout a 24 h cycle in most research concerning leaf *R*.

The underlying concept is that diel variation in *R* at any T (*R*_T_) is typically described based on two components (i) a constant rate of *R*_T_ (*R*_To_, Supplementary Table 7) at an arbitrarily set T, T_o_, and (ii) the T-sensitivity of *R*, as: ${R}_{T}={R}_{{T}_{o}}{{Q}_{10}}^{[(T-{T}_{o})/10]}$ (Equation 1, termed here the standard model), where Q_10_, the T-sensitivity, is the relative change in *R* obtained with a 10 °C change in T. Following ref. 22., Q_10_ per se also varies with measured T, so that the temperature-dependent Q_10_ (TDQ_10_), ${Q}_{10}(T)=3.09-0.0435 { \times \; T}$ (Equation 2) can replace Q_10_ in Equation 1.

In addition to T-changes, *R* may, however, also be controlled by endogenous factors, such as availability of substrates^23,24^, use of respiratory products^23–26^, and the relative engagement of enzymes, e.g. the alternative oxidase, in plants^27,28^, all of which exhibit diel variation. Diel variation can be caused by circadian rhythms in gene expression of many enzymes^29^ potentially affecting *R*, or by changes in environmental cues, substrate availability, or demand for respiratory substrate. The extended night-time period of substrate use and translocation in the absence of photosynthesis is therefore likely to exhibit systematic changes in *R*.

Despite the current consensus of a constant *R*_To_ in estimates of 24 h integrals of CO_2_-efflux underlying multiple types of biological models, we hypothesise that there is a nocturnal variation in *R*_To_ that is independent of short-term temperature control of metabolic rates (i.e. non-temperature control)^30,31^. Therefore, it is essential that we evaluate the degree to which temperature controls diel variation in plant *R*. If the temperature does not control the full diel *R* variation, we need to quantify the temporal, temperature-independent variation in *R*_To_. At the same time, for the part of temporal variation in *R* that is controlled by temperature, it is important that we understand whether estimates of temperature sensitivity (Q_10_) of *R* may be dependent on the duration of measurements, due to unintentional yet potential inclusion of temporal variation in *R*_To_ in the calculation of Q_10_. Typically, nocturnal leaf *R* is estimated from so-called dark-adapted leaf *R*, measured during the daytime^21^, but in our study, we examined the phenomenon of ‘non-temperature control’ of respiration by focusing on nocturnal leaf *R* as respiratory CO_2_-release. We use available literature data and new measurements from both the lab and the field to evaluate whether *R*_To_ is a constant or variable through the night and create an empirical model that represents a nocturnal variation of *R*_To_. The model is evaluated using an independent data set collected in the field under variable temperature conditions. We then illustrate the importance of the difference between a constant and a variable *R*_To_ in global estimates of plant CO2 efflux to the atmosphere. The focus of this study is solely on the effect of ‘non-temperature control’ of plant respiration and we do not address longer-term thermal acclimation. We make use of data of *R*_To_ measured in fully expanded leaves. Therefore, nocturnal variation in *R*_To_ in this study in theory represents only the maintenance part of respiration^2^.

Here, we show that the rate of leaf respiration at constant temperature decreases monotonically with time through the night, on average, by 25% after 8 h of darkness. Temperature controls less than half of the total nocturnal variation in respiration. A new universal formulation is developed to model and understand nocturnal plant respiration, combining the nocturnal decrease in the rate of leaf respiration at a constant temperature with the decrease in plant respiration according to the temperature sensitivity. Application of the new formulation within a terrestrial biosphere model shows a global reduction of 4.5−6% in plant respiration and an increase of 7–10% in net primary production for the present-day, with the largest effects in the tropics.

## Results

### Is *R*_To_ constant at night?

We searched all available literature for measurements of leaf *R*_To_ (as CO_2_-efflux) when measured more than once within a period of darkness (simulating night-time) in lab-based studies where the measurement temperature, T_o_, was kept constant (Supplementary Table 1, 15 species). From these experimental data for each species, we plotted *R*_To_ throughout the night normalised to the initial measurement of *R*_To_ (*R*_To-initial_, i.e. *R*_To_ measured at onset of darkness, Supplementary Fig. 1a). For each species, *R*_To_/*R*_To-initial_ decreased during the night at constant T_o_ and could be described by a monotonic power- or linear function (Supplementary Fig. 1a).

We further evaluated whether the monotonic decrease of *R*_To_/*R*_To-initial_ from lab experiments is observable in the field. *R*_To_/*R*_To-initial_ measured during the night from our field data was complemented with published data from the field, where leaf *R*_To_ also was measured more than once during night-time at constant T_o_ (Supplementary Table 1, 16 species). All species in the field exhibited monotonic power or linear decreases in *R*_To_/*R*_To-initial_ during the night-time (Supplementary Fig. 1b).

**Fig. 1:**
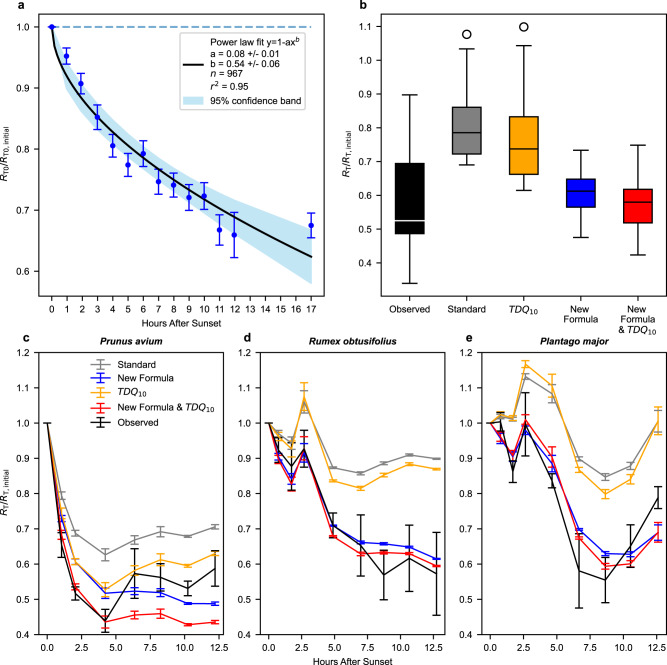
Nocturnal variation in the rate of respiration. **a** Average power function (full line + confidence interval in blue, *R*_To_/*R*_To-initial_ = 1–0.08 × h^0.54^, *r*^2^ = 0.95) of decrease in nocturnal leaf *R*_To_/*R*_To-initial_ (Supplementary Table 1, 5) measured at constant T_o_ under field (16 species) and lab (15 species) conditions. Each point in the plot represents a mean value of 4–92 replicate individuals per hour, across all species (±SEM). *n* = total of 967 nights of leaf *R*_To_/*R*_To-initial_ across 31 plant species. Null-hypothesis of *R*_To_/*R*_To-initial_ = 1 (as in Equation 1) is shown by a dashed line. *R*_To_ is leaf respiration at a constant temperature, T_o_. Initial defines the first measurement. **b** Box-and-whisker plots (The centre line is the median. The lower whisker is the lowest datum above the first quartile − 1.5 × interquartile range. The upper whisker is the highest datum below the first quartile − 1.5 × interquartile range. Any points outside the whiskers are plotted separately) (*n* = 9 species) observed and modelled *R*_T_/*R*_T-initial_ in nine field-grown broad-leaf species (Supplementary Fig. 2, one to six replicate individuals per species) at 8–13 h after sunset. Modelled (Suppl Table 4) values are Standard (Equation 1 and Q_10_ = 2), Standard modified (Equation 1 and TDQ_10_ (i.e. temperature-dependent Q_10_, where Q_10_ is the relative change in *R* obtained with a 10 °C change in T), New formulation (Equation 4 and Q_10_ = 2), and New formulation modified (Equation 4 and TDQ_10_). *R*_T_ is leaf respiration at varying temperatures, T. **c** Average (±SEM) observed- and modelled temporal nocturnal development of leaf *R*_T_/*R*_T-initial_ of *Prunus avium* (Supplementary Fig. 2b–d, *n* = 4). **d** Average (±SEM) observed- and modelled temporal nocturnal development of leaf *R*_T_/*R*_T-initial_ of *Rumex obtusifolius* (Supplementary Fig. 2b–d, *n* = 4). **e** Average (±SEM) observed- and modelled temporal nocturnal development of leaf *R*_T_/*R*_T-initial_ of *Plantago major* (Supplementary Fig. 2b–d, *n* = 4). Data were available in Supplementary Data 1–3.

Welch two-sample *t*-tests showed the slopes of linearised relationships describing the nocturnal decrease in *R*_To_/*R*_To-initial_ did not significantly differ between lab and field (mean of slopes tested against each other: lab = −0.222 and field = −0.174, *p* = 0.29, *t*-test, df = 21), between tree and herbaceous species (mean of slopes tested against each other: tree = −0.164 and herbaceous = −0.215, *p* = 0.18, *t*-test, df = 27), nor between species originating from temperate or from tropical biomes (mean of slopes tested against each other: temperate = −0.227 and tropical = −0.175, *p* = 0.28, *t*-test, df = 20). Therefore, a universal model across all 31 examined species could be produced, *R*_To_/*R*_To-initial_ = 1 – 0.08 × h^0.54^ (Equation 3, number of nights = 967, *r*^2^ = 0.95, Fig. 1a) with h defined as the time (in hours) since the onset of darkness (lab) or sunset (field) describing the mean temperature-independent decrease of *R*_To_/*R*_To-initial_ during night-time. Based on this universal model across species, on average *R*_To_/*R*_To-initial_ decreases by 25% (±1.8%, 95% CI) after 8 h of darkness.

### New formulation of temporal variation in nocturnal plant respiration

We merged the current representation of the temperature response of *R* from both Equation 1 and Equation 2 with our new finding of Equation 3, that represents non-temperature control of nocturnal respiration. This created new formulations that represent the temporal variation of *R*_T_ at any time (*t*) during night-time in response to both a varying T and the nocturnal temperature-independent decrease in *R*_To_. The merging of e.g. Equation 1 and Equation 3 yields *R*_T,*t*_ = *R*_T,sunset_ × Q_10_^0.1×(T,t – Tsunset)^ × (1 – 0.08 × h^0.54^) (Equation 4) with *R*_T,t_ and T,t defined as *R*_T_ and T at timestep t, respectively, and *R*_T,sunset_ defined as *R*_T_ at sunset. The corresponding merging of Equation 2 and Equation 3 yields *R*_T,*t*_ = *R*_T,sunset_ × TDQ_10_^0.1×(T,t – Tsunset)^ × (1 – 0.08 × h^0.54^) (Equation 5). We evaluated the validity of this new model of *R*_T,*t*_ with field data collected during night-time under ambient conditions with natural T variation for another ten species (see Methods). Simulated *R*_T_ using this new formulation (Equations 4, 5) represents the observations of *R*_T_ at varying T and time more successfully than with the standard model of Equation 1 or with Equation 2 at both leaf levels (Fig. 1b, c and Supplementary Fig. 2a–d for nine species) and whole-tree scale (Supplementary Fig. 3a–d for one species).

### Temperature control of nocturnal respiration

**Fig. 2:**
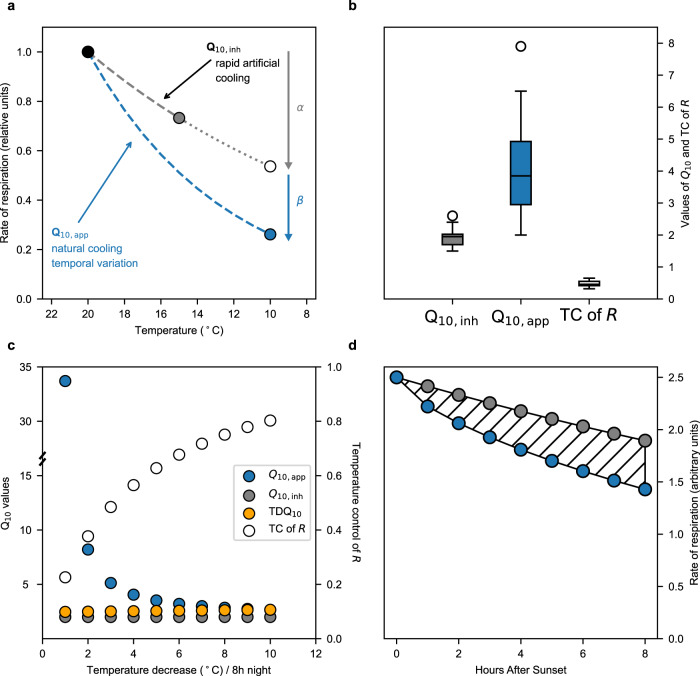
Concepts explained. **a** Conceptual figure of how *R*_T_ (rate of respiration at varying temperature) is measured in response to either (i) common short-term rapid artificial cooling (e.g. during 10–30 min) of leaf/plant can be used to calculate Q_10,inh_ (inherent temperature sensitivity of *R*, Equation 1, using point connected by the grey dashed line), or (ii) natural cooling (over several hours) of the environment along temporal variation in T can be used to calculate Q_10,app_ (apparent temperature sensitivity of *R*, Equation 1, using the points connected by the blue dashed line). The decrease in *R*_T_ due to change in T alone is denoted α and the further observed decrease in *R* is denoted β. Common practise is also to extrapolate (represented by a short-dashed curve) how *R*_T_ is assumed to change (open symbol) with T-change further into the night than the actual measured T-change (long dashed curve). **b** Box-and-whisker plots (The centre line is the median. The lower whisker is the lowest datum above the first quartile − 1.5 × interquartile range. The upper whisker is the highest datum below the first quartile − 1.5 × interquartile range. Any points outside the whiskers are plotted separately) of Q_10,app_, Q_10,inh_, and TC (temperature control) of *R* (α/(α + β)) across ten species (46 replicate plants, Supplementary Table 2). **c** Dependence of Q_10_ and TC of *R* on the rate of nocturnal cooling. In the example shown, it was assumed Q_10,inh_ = 2.0. Q_10,app_ and TC were calculated as described in Fig. 2a assuming that *R*,_To_/*R*,_To-initial_ = 1–0.08 × h^0.54^ (Fig. 1a). TDQ_10_ = 3.09 − 0.0435 × T (temperature-dependent Q_10_, ref. 22, Equation 2). **d** Modelled nocturnal variations in *R* in response to T decrease during the night, including and excluding effects of non-temperature control on metabolism. *R*_T_ = *R*_To_ × Q_10,inh_^[(T-To)/10]^, where *R*_To-initial_ = 2.5 and Q_10,inh_ = 2. Grey symbols represent constant *R*_To_ and a T decrease of 0.5 °C/h. Blue symbols represent *R*_To_/*R*_To-initial_ = 1 – 0.08 × h^0.54^ (Fig. 1a) and a temperature decrease of 0.5 °C/h. Dashed area is the cumulated difference in nocturnal *R* with time throughout the night without and with non-temperature control. Data are available in Supplementary Data 4.

Given that *R*_To_/*R*_To-initial_ < 1 throughout night-time (Fig. 1a) and that Equations 4, 5 explain temporal variation in *R*_T_ better than the standard model, i.e. Equation 1 (Fig. 1b, c and Supplementary Figs. 2a–d,  3a–d), it is implied that *R* is not under full temperature control (TC) on a nocturnal basis as previously widely assumed^2–5,10^. We, therefore, examined the degree to which *R* is under TC on a nocturnal basis as TC of *R* = α/(α + β) over a given time period, where α is the decrease of *R*_T_ due to T-change alone and β is the further decrease due to any temperature-independent temporal changes (Fig. 2a). We measured both α and β in the field (Supplementary Table 2, ten species and for one species also during different seasons) and found that TC of *R* was only 0.48 ± 0.05 (mean ± SE) out of 1 (Fig. 2b).

As TC of *R* < 1 (Fig. 2b), it is further implied that there should be a systematic difference between apparent temperature sensitivity of *R* (Q_10,app_) measured under ambient conditions over the course of the night (through several hours), i.e. including the factor of time, and inherent temperature sensitivity (Q_10,inh_, Equation 1) measured in response to a brief T-manipulation over the course of minutes (Fig. 2a). Thus, by definition Q_10,inh_ is the closest we get to represent a true temperature sensitivity, and it reflects α with a given change in T (Fig. 2a). In contrast, Q_10,app_ is defined as only an apparent temperature sensitivity because it can be calculated from changes in *R*_T_ and changes in T (Equation 1), but it includes in reality also temporal changes to *R*_To_ due to non-temperature effects (β, Fig. 2a) and therefore also further changes to the measured *R*_T_. We compared all available studies where both Q_10,inh_ and Q_10,app_ of leaf *R* was measured (own lab- and field-based experiments and published literature, Supplementary Table 2) and found in the ten examined species that Q_10,inh_ was less than half of Q_10,app_, (Fig. 2b).

In consequence, if the speed of cooling during the night is low, TC of *R* is low and both Q_10,inh_ (Equation 1) and TDQ_10_ (Equation 2) are far from predicting the realised Q_10,app_ (Fig. 2c). Ignoring nocturnal variation of *R*_To_ compared to *R*_To,initial_ as done in the standard model, leads to an overestimation of accumulated nocturnal respiratory CO_2_-efflux (Fig. 2a, d); this overestimation increases with the duration of night-time and with lower TC of *R* (Fig. 2c–d and Supplementary Fig. 4).

### Global plant *R* and *NPP* when accounting for nocturnal variation in *R*_To_

**Fig. 3:**
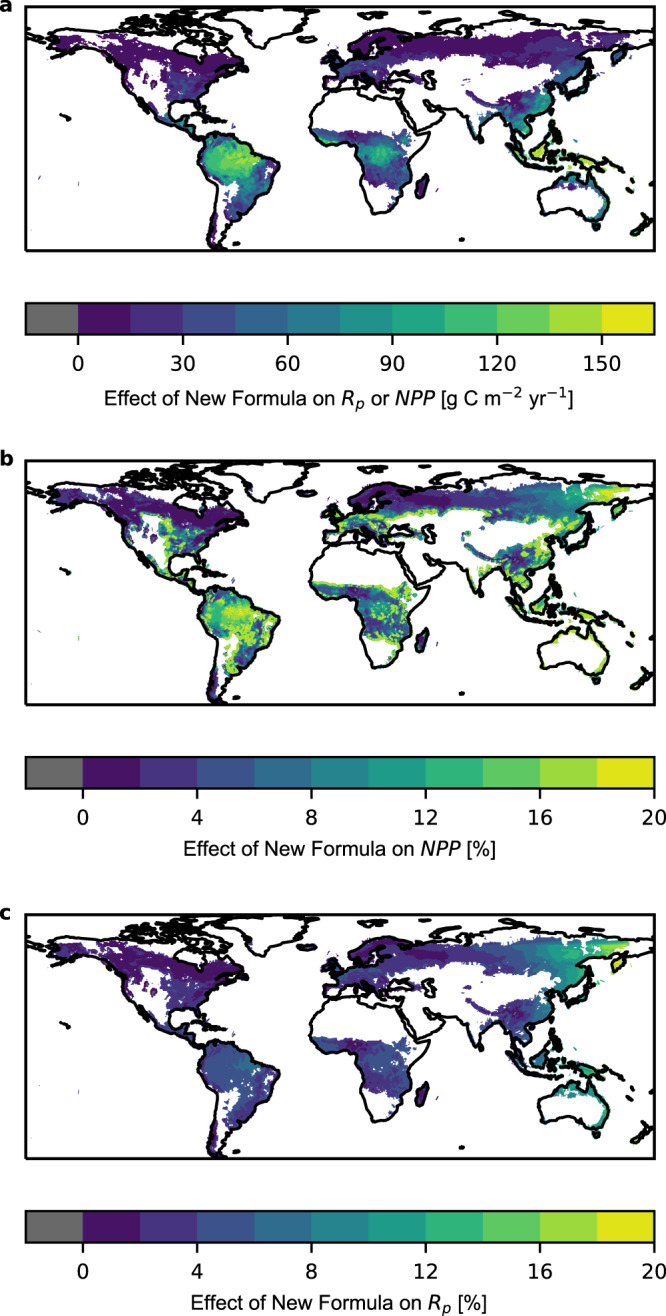
Global modelling of *R*_P_ and *NPP*. Impact of incorporation of nocturnal variation in whole plant *R*_To_ in a simulated reduction in plant respiration *R*_p_ (**a**, **c**) and a corresponding increase in NPP (**b**) over the period 2000–2018 using the standard model with *Q*_10_ = 2 (Equation 1) and the new formula (Equation 4). The impact is estimated as the difference between the temporal mean of simulations with and without nocturnal variation in whole plant *R*_To_ (respiration rate at constant temperature) for *NPP* (net primary production) and vice versa for *R*_p_ (plant respiration rate) (**a**) and as a percentage of simulations without nocturnal variation in *R*_To_ (**b**, **c**). Note, that the reduction in *R*_p_ (**a**) is identical to the increase in NPP in absolute terms. Results are presented for grid cells where grid level *NPP* is >50 g m^−2^ yr ^−1^ in the standard simulations with Q_10_ = 2 (the relative change in *R* obtained with a 10 °C change in T) to avoid excessively large % effects at very low NPP.

The implications of considering nocturnal variability of *R*_To_ on regional and global patterns of plant respiration (*R*_p_) and net primary production (*NPP*) was assessed using a global TBM, the Joint UK Land Environment Simulator (JULES^32,33^), which is the land surface model of the UK Earth System Model. We incorporated the new formulation (Equations 4, 5) that accounts for both TC and non-TC of *R* into JULES and compared it against predictions of *R*_p_ and *NPP* using the standard (Equation 1) and the TDQ_10_ (Equation 3) formulations that only account for TC of *R*. Implementation of Equation 4, 5 in JULES is based on our findings of nocturnal variation in leaf *R*_To_ (Fig. 1a), and the assumption that the whole plant (leaves, canopy, roots and stems) *R* also exhibits nocturnal variation in *R*_To_^30,31,34–36^ (Supplementary Fig. 3). Incorporation of nocturnal variation of *R*_To_ into JULES results in a decrease in simulated *R*_p_ globally of 4.5–6% (5−6% with TDQ_10_) and an increase on simulated *NPP* of 8–10% (7–9 % with TDQ_10_) (Fig. 3; for simulations including TDQ_10_ see Supplementary Fig. 5). This effect is mostly driven by the tropics (here defined as latitudes between 30°N and 30°S) where the impact on *R*_p_ is a decline of 5–6% (5–7% with TDQ_10_) and an increase in *NPP* of 9–11.5% (9–11% with TDQ_10_). Quoted percentage ranges of effects (Supplementary Table 6) include upper and lower confidence intervals derived from Equation 3 (Supplementary Table 4).

## Discussion

Our results demonstrate that the common assumption of a constant rate of respiration at a constant temperature, *R*_To_, during night-time conflicts with the measured *R*_To_/*R*_To,initial_ (as CO_2_-efflux), which decreases during the night-time (Fig. 1a and Supplementary Fig. 1a + b). In further support, Q_10,inh_ is less than half of Q_10,app_ (Fig. 2b and Extended Table 2), which only can happen if *R*_To_/*R*_To,initial_ decreases during the night-time (Figs. 1a,  2a).

To account for non-temperature control and to include nocturnal variation in *R*_To_, we propose a new equation. This equation predicts temporal variation in *R*_T_, representing an improvement of the standard Equation 1 or Equation 2 currently used in TBM’s and Earth System Models (Fig. 1b, c and Supplementary Figs. 2a–d,  3a–d). In support of plant level applicability of this model equation, we found that there are previous indications of temporal variation in respiratory CO_2_-efflux (however, without knowledge of potential contribution from growth respiration) when measured at the same T of canopies (nocturnal decrease by 25% in *Phaseolus vulgaris*; nocturnal decrease by 62% in *Gossypium*)^35^, root + soil (diel variation in by 35% at T_soil_ = 19 °C)^30^, stems (nocturnal decrease by 17% in *Hymenolobium pulcherrimum*)^34^ and at an ecosystem level, i.e. soil and plant *R*, (nocturnal decrease by 25% after 8 h night-time in a coniferous temperate forest, Howland Maine, USA)^37^.

There was no significant difference in *R*_To_/*R*_To-initial_ as power functions of time of night between lab conditions with constant plant-T_o_ and field studies where only leaf-T_o_ was kept constant (Supplementary Fig. 1a, b). Therefore, *R*_To_/*R*_To-initial_ < 1 at night is not confounded by decoupling leaf-T from plant-T^38^. Non-temperature control in diurnal variation of plant photosynthesis and stomatal conductance has been reported, with circadian rhythms responsible for 15–25% and for 30–35% of the daytime oscillations, respectively^39^. In comparison, the non-temperature control component is responsible for, on average, 52% of the night-time variation in *R* in this study (Supplementary Table 2), implying that the control of temporal variation in leaf *R* at night is approximately equally divided between T-changes via the inherent T-sensitivity (Equation 1) and other non-temperature-control factors affecting *R*_To_ (Equation 3). However, TC may change between seasons and between biomes due to both changes in the speed of nocturnal cooling (Fig. 2a) and length of night (Supplementary Fig. 4). Therefore, a temporal variation of non-temperature control of *R*_To_ should be accounted for in all future modelling of nocturnal plant *R* and integrals of respiratory CO_2_-release.

Plant *R* and its T-sensitivity have been measured and reported for more than a century^40^. However, never has there been such a systematic focus on the temporal component on night-time variation in *R*_To_ when estimating and analysing T-sensitivity (Fig. 2a). This may explain the hitherto conceptual confusion of Q_10,inh_ and Q_10,app_^2,22,41,42^, where the distinction has not been made between T-sensitivity with or without a significant confounding temporal component (Fig. 2a). In addition, it has for some time been assumed that the TDQ_10_ (Equation 2), replacing Q_10_ in Equation 1 might reasonably be applied to predict temporal variation in *R*_T_^22,41,43,44^. However, as shown in Fig. 2c TDQ_10_ does not resemble the realised Q_10,app_, especially if the speed of nocturnal cooling of the environment is low and the night-time decline in base respiration rate dominates the observed pattern in respiration. Indeed, the use of TDQ_10_ with Equation 1 does not predict temporal variation in *R*_T_ as well as when the TDQ_10_ is used in combination with our new formulation (Equation 5) (Fig. 1b, c and Supplementary Fig. 2a–d).

Physiological studies examining differences in nocturnal plant *R* between species, developmental stages, organs, environmental conditions, and seasonal variation may be biased if not examined (i) at night and (ii) at the same time of the night. Thus, estimates of leaf *R* in the dark from daytime measurements at varying times of day (e.g. GlobResp^20,21^) may not reflect night-time rates of foliar respiration in the dark. Our study only focused on night-time as daytime leaf *R* is potentially inhibited by light^45–47^. However, we consider it very important to gather data to test the hypothesis of daytime variation of *R*_To_.

Incorporating a nocturnal decrease in plant *R*_To_ into a TBM suggests that global *R* has been previously overestimated and global *NPP* has been underestimated, especially in tropical regions, which have the lowest speed of cooling overnight and longest nights on average over the course of the year (Fig. 2c). This has significant implications for both empirical and modelling studies that focus on ecosystem-level processes since most observations used to calibrate and or evaluate the models miss this process and different methods that ignore this process have been compared against each other^14^.

Within the plant growth-and-maintenance-respiration paradigm (sensu Amthor)^2^, where growth *R* is considered temperature-insensitive and maintenance *R* is temperature-sensitive, our data (Fig. 1a) may be interpreted as evidence for a general nocturnal decreasing trend in leaf growth *R* during the night, which needs to be taken into account in plant respiration models (Equation 3). However, respiration supports biochemical reactions that are difficult to categorise into growth and maintenance, and within the *general paradigm* (s*ensu* Amthor)^2^, we still lack knowledge of potential general trends of nocturnal variation in both rates of processes supported by respiration and their metabolic costs^2^. One conclusion from our data (Fig. 1a) with respect to the plant growth-and-maintenance-respiration paradigm (sensu Amthor)^2^ is that in future measurements of the nocturnal temperature-sensitivity (Q_10,inh_) of leaf maintenance *R* alone (i.e. without growth *R*) using Equation 1, researchers need to be aware of the presence of biochemical reaction supported by *R* that may decline steadily at night as the ratio of *R*_T_/*R*_To_ for a given T/T_o_ most likely does not reflect only the temperature sensitivity of maintenance *R*, which is commonly assumed. Therefore, a future challenge is to understand how this non-temperature control of *R* may be distributed among diel variation in availability of respiratory substrates (sugars from photosynthesis^23,24,48^), the demand for respiratory products (e.g. ATP^23–26,48^), the relative engagement of AOX^27,28^, other de-carboxylation processes, and how this might vary among species, growth forms, and environmental conditions.

Because circadian and diurnal rhythms are found in all examined eukaryotes^49^, we expect the new equation (Equation 4) to have implications for the many scientific disciplines mentioned above.

## Methods

### Literature values of *R*_To_ and Q_10_ of leaf respiration

Data of *R*_To_ were read from texts, tables, and figures in all available literature (18 species; Supplementary Tables 1, 2) when measured more than once within a period of darkness in lab- and field studies where measurement temperature, T_o_, was kept constant. The *R*_To-initial_ was defined as the initial measurement of *R*_To_ for each study/species, and further values of *R*_To_ at later points within the same night of the same study were read as well.

Apparent- and inherent temperature sensitivities (Q_10_, Equation 1; Fig. 2b) were obtained from all available literature (ten species; Supplementary Table 2) where in the same study/species, both nocturnal values of Q_10,app_ and of Q_10,inh_ were obtained in response to long-term natural T-changes in the environment during the night (hours) and nocturnal values were obtained in response to short-term artificial T-changes (max 30 min), respectively.

### Measurements of *R*_To_ and Q_10_ of leaf respiration

In the field (United Kingdom, Denmark, Panama, Colombia and Brazil), *R*_To_ (µmol CO_2_ m^−2^ s^−1^) in 16 species (Supplementary Tables 1, 3) was measured through nocturnal periods at constant T_o_ (controlled either by block-T or leaf-T) with infra-red gas analysers (Li-Cor-6400(XT) or Li-Cor-6800, Lincoln, Nebraska, USA). Mature, attached leaves positioned in the sunlight throughout the day were chosen. Target [CO_2_] in the leaf cuvette was set to ambient, ranging from 390 to 410 ppm, depending on when measurements were made, and target RH = 65 ± 10%, with a flow rate of 300 µmol s^–1^. The *R*_To-initial_ was defined as *R*_To_ at first measurement after darkness 30 min after sunset (to conservatively avoid light-enhanced dark respiration, LEDR^50,51^. Leak tests were conducted prior to measurements^52^. The temporal resolution of measurements varied between every three minutes to once per hour for the different species. Data were subsequently binned in hourly bins.

Measurements to derive Q_10,inh_ and Q_10,app_ were conducted in two species in a T-controlled growth cabinet and in six species in the field (Supplementary Table 2), where Q_10,inh_ was measured in response to 10–30 min of artificial changes in T and Q_10,app_ was calculated from measurements of *R*_T_ in response to T of the environment (growth cabinet or field) at the beginning of the night and again at the end of the night (hours apart).

### Tree level measurements in whole-tree chambers

The night-time respiratory efflux of the entire above-ground portion (crown and bole) in large growing trees of *Eucalyptus tereticornis* was measured in whole-tree chambers (WTCs) in Richmond, New South Wales (Australia, (33°36ʹ40ʺS, 150°44ʹ26.5ʺE). The WTCs are large cylindrical structures topped with a cone that enclose a single tree rooted in soil (3.25 m in diameter, 9 m in height, volume of ~53 m^3^) and under natural sunlight, air temperature and humidity conditions. An automated system measured the net exchange of CO_2_ between the canopy and the atmosphere within each chamber at 15-min resolution. During the night, we used the direct measurements of CO_2_ evolution (measured with an infra-red gas analyser; Licor 7000, Li-Cor, Inc., Lincoln, NE)^53,54^ as a measure of respiration.

Due to the high noise-to-signal ratio in the CO_2_-exchange measurements from this system when analysing the high-resolution temporal variation through each night, we chose to only analyse temporal variation in tree-*R*_T_ for the nights when tree-*R*_T-initial_ were amongst the top 10% of CO_2_-exchange signals for the entire data set. The resulting data spanned 62 nights and included hourly average measurements from three replicate chambers.

### Data analysis of *R*_To_

Measurements of nocturnal leaf respiration under constant temperature conditions (*R*_To_) were divided by the initial rate of respiration (*R*_To-initial_) at the onset of each night. Hourly means of *R*_To_/*R*_To-initial_ were calculated for each leaf replicate to remove measurement noise and reduce bias due to the measurement of some species at more frequent intervals throughout the night. For species with multiple leaf replicates, these hourly means of *R*_To_/*R*_To-initial_ were then combined to create hourly averages of *R*_To_/*R*_To-initial_ at the species level. For each species, these values were plotted as a function of time to demonstrate how *R*_To_/*R*_To-initial_ decreases with time since the onset of darkness, from sunset until sunrise (Supplementary Fig. 1). For each species, hourly means of *R*_To_/*R*_To-initial_ plotted as a function of time were linearised by log-transforming data and the slope of the relationship determined. To test whether the slopes of the lines differed significantly within plant functional groups (woody, non-woody), species originating from the same biome (temperate, tropical) or species measured under the same conditions (lab, field), the slopes of the lines for all species from a given functional group, biome or measurement condition were tested pairwise against each other using the slope, standard error and sample size (number of points on the x-axis) for each line and applying a 0.05 cut-off for *p* values after Bonferroni correction for multiple testing. 11 out of 701 comparisons came out as being significantly different, which is why within-group slope differences were considered to be overall non-significant for this analysis. *t*-tests were used to test whether the slopes differed between plant functional groups (tree, non-woody), species originating from different biomes (temperate, tropical) and species measured under different environmental conditions (lab, field). In these tests, the degrees of freedom varied according to the different sample sizes. Since *R*_To_/*R*_To-initial_ plotted as a function of time always starts at 1, the intercepts do not differ between species. *t*-tests were performed on linearised power functions by log-transforming data in order to test potential differences between lab and field, origin of species, between woody and non-woody species and between temperate and tropical biomes. Since these functions were statistically indistinguishable in each pairing, all measurements of nocturnal leaf respiration under constant temperature conditions (*n* = 967 nights, 31 species) were collated into a single plot. The data were binned hourly since some studies had very few measurements on half-hourly steps. A power function was fitted with a weighting of each hourly binned value using 1/(standard error of the mean). The power function was chosen as it, better than the exponential- or linear function, can capture both sudden steep- as well as slower decrease in *R*_To_/*R*_To-initial_ in different species. The 95% confidence interval of the power function, following the new model equation, overlaps with all the 95% confidence intervals of the hourly binned values (Fig. 1a). All data analysis, including statistical analysis and figures were performed using Python version 3.9.4.

### Evaluation of new equation

We performed four sets of simulations (S1-S4) using different representations of leaf and plant respiration as outlined in Supplementary Table 4. Evaluation of Equation 4 (S2; Equation 3 from Fig. 1a merged with Equation 1) in comparison with Equation 1 (S1) and Equation 5 (S4) in comparison with Equation 2 (S3), respectively, for predictions of nocturnal variation in response to natural variation in temperature, was conducted by use of independent sets of leaf level data and tree scale data. The effect of including variable nocturnal *R*_To_ is estimated as the difference between S1 and S2 and between S3 and S4, respectively.

The first data set used for the evaluation consists of nine broad-leaf species for which spot measurements of leaf respiration under ambient conditions were taken at sunset and before sunrise in the field (Fig. 1b and Supplementary Fig. 2a). Of these nine species, three species (Fig. 1c) were further measured throughout the night at ambient conditions. Further, whole-tree measurements measured throughout the night at ambient conditions (Supplementary Fig. 3a–d) were also used for evaluation. Finally, comparisons of Q_10,inh_ with Q_10,app_ in another ten species were used to test if *R*_To_ appeared constant as assumed in Equation 1 (Supplementary Tables 2, 3 and Fig. 2b).

To validate the suitability of Equation 4 and Equation 5 over equations with full temporal control, modelled respiration values were compared against observed measurements for three species at the leaf level (Supplementary Fig. 2b–d) and for *Eucalyptus tereticornis* at the whole-tree level using three chamber replicates and during 62 nights using hourly measurements (Supplementary Fig. 3a–d). Linear fits were applied, using ordinary least squares regressions, to plots of normalised respiration (${R}_{T}/{R}_{{T}_{0}}$) predicted by the four models against the observed values. The first measurements of the night were excluded from the fits, as these were necessarily equal to unity. The standardised residuals (*S*) in Supplementary Figs. 2c, 3b are calculated using the equation ${S}_{i}=({R}_{{{{{{{\rm{modelled}}}}}}}_{i}}/{R}_{{{{{{{\rm{Modelled}}}}}}}_{0}}-{R}_{{T}_{i}}/{R}_{{T}_{0}})/\sqrt{(\mathop{\sum }\nolimits_{i}^{N}{({R}_{{{{{{{\rm{modelled}}}}}}}_{i}}/{R}_{{{{{{{\rm{Modelled}}}}}}}_{0}}-{R}_{{T}_{i}}/{R}_{{T}_{0}})}^{2})/{df}}$, for the residual of the *i*th measurement, where the sum is over all measurements, *df* is the number of degrees of freedom, and R_modelled_ are the respiration values modelled by the four equations in Supplementary Table 4.

Evaluation is done by comparing observed and simulated *R*_T_/*R*_T, initial_. We evaluate the nocturnal evolution of *R*_T_/*R*_T, initial_ and use (i) one-to-one line figures that include fitted regression line, *R*^2^, *p* value and RMSE, (ii) Taylor diagrams and (iii) use plots of standardised residuals against temperature and hours since darkness for a qualitative assessment of the simulations, to identify whether there are any model biases at specific times or temperatures. Model evaluation, statistical analysis and figures were done using python version 3.9.4.

### Global scale modelling of plant *R* and *NPP*

We applied the novel formulation derived in this study (Equation 4 and Equation 5) to quantify the impact of incorporating variable *R*_To_ on simulated plant *R* and *NPP* globally using the JULES land surface model^32,33^ following simulations outlined in Supplementary Table 4.

Plant respiration in JULES and simulations for this study: The original leaf respiration representation in JULES follows either eqn 1 ${{R}_{T}={R}_{{T}_{0}}{Q}}_{10}^{(T-{T}_{0})/10}$ with Q_10_ = 2 and T_o_ = 25 ^o^C or Equation 1 with an additional denominator ${{R}_{T}={R}_{{T}_{0}}{Q}}_{10}^{(T-{T}_{0})/10}/\left\lfloor \left(1+{e}^{0.3(T-{T}_{{upp}})}\right)\times \left(1+{e}^{0.3({T}_{{low}}-T)}\right)\right\rfloor$ (Equation 6). For the purpose of this application, we have used Equation 1 to represent leaf respiration in standard JULES simulations. The remaining components of maintenance respiration in JULES, i.e. fine root and wood are represented as a function of leaf to root and leaf to wood nitrogen ratios and leaf respiration rates following *R*_T_ (*β* + (*N*_r_ + *N*_s_)/*N*_l_) (Equation 6) with *R*_T_ as leaf respiration, *N*_r_, *N*_s_ and *N*_l_ as root, stem and leaf Nitrogen content respectively and *β* as a soil water factor (Equation 42 in ref. 32). This implies that any variation in leaf respiration is passed to root and wood respiration as well^30,31,35^. Growth respiration is estimated as a fraction (25%) of the difference between GPP and maintenance respiration (*R*_m_) expressed as *R*_g_ = 0.25 (*GPP*-*R*_m_).

JULES version 5.2 was modified to simulate leaf and plant respiration using the various descriptions (Equations 1–5) outlined in the modelling protocol in Supplementary Table 4. JULES uses standard astronomical equations to calculate the times of sunrise and sunset on a given day at each grid point. We used the model leaf temperature and *R*_T_ at the timestep at or immediately preceding sunset to represent T_sunset_, and *R*_T,sunset_ and at every timestep through the night, the time since sunset (h) was updated. We performed global simulations for the period 2000–2018 with JULES, using the global physical configuration GL8, which is an update from GL7^55^. We used WFDEI meteorological forcing data^56^ available at 0.5-degree spatial resolution and 3-h temporal resolution, and disaggregated and run in JULES with a 15 min timestep. Simulations were performed using nine plant functional types (PFTs)^33^. To isolate the effects of the new formulation on simulated *R*_p_ and *NPP* from possible impacts on leaf area index (LAI) or vegetation dynamics, we prescribed vegetation phenology via seasonal LAI fields and vegetation fractional cover based on the European Space Agency’s Land Cover Climate Change Initiative (ESA LC_CCI) global vegetation distribution^57^, processed to the JULES nine PFTs and re-gridded to the WFDEI grid. Annual variable fields of CO_2_ concentrations are based on annual mean observations from Mauna Loa^58^. JULES was spun up using the three cycles of the 2000–2018 meteorological forcing data to equilibrate the soil moisture stores. The mean annual output of *R*_p_ and *NPP* over the study period (2000–2018) is computed for all simulations and the effect of the new formulation is presented as the difference between the temporal mean of simulations with and without nocturnal variation in whole plant *R*_To_ for *NPP* and vice versa for *R*_p_ and as percentage respect to simulations without nocturnal variation in *R*_To_. Results are presented for grid cells where grid level *NPP* is >50 g m^−2^ yr ^−1^ in the standard simulations to avoid excessively large % effects at very low *NPP*. Output from JULES was analysed and plotted using python version 2.7.16.

### Permits

No permit was required in Denmark as measurements were taken in private land (of author) and public land and measurements were non-destructive. Data were collected under the Panama Department of the Environment (current name MiAmbiente) research permit under the name of Dr Kaoru Kitajima. Permit number: SE/P-16-12. Data in Brazil were collected under the minister of Environment (Ministério do Meio Ambiente—MMA), Instituto Chico Mendes de Conservação da Biodiversidade—ICMBio, Sistema de Autorização e Informação em Biodiversidade—SISBIO permit number 47080-3. No permit was required in Colombia as measurements were taken on private land, no plant samples were collected, and trees were part of an existing experiment for which one of the co-authors is the lead. No access permits were required in the UK as they were conducted on the campus of own university plus in their own private garden.

### Reporting summary

Further information on research design is available in the Nature Research Reporting Summary linked to this article.

## Supplementary information

Supplementary Information

Description of Additional Supplementary Files

Supplementary Data 1

Supplementary Data 2

Supplementary Data 3

Supplementary Data 4

Supplementary Data 5

Reporting Summary

## Data Availability

The leaf respiration data measured as part of this study and collected from the literature together with annual gridded JULES output generated in simulations of this study are available at 10.5281/zenodo.7037530. WFDEI meteorological forcing data is available at the DATAGURU website for climate-related data at Lund University (https://DATAGURU.lu.se, then go to “Explore available datasets”). This allows extraction of data from the global domain, a user-defined grid box or region for a specified time interval. Ftp downloads are possible via the unix/linux command line, site = ftp.iiasa.ac.at, username = rfdata and password = forceDATA, this takes the user to the WATCH Forcing DATA files, then switch to the WFDEI directory using: ‘cd WFDEI’. The /WFDEI directory includes files listing grid box elevations and locations Annual CO_2_ concentrations are available at https://gml.noaa.gov/ccgg/trends/gl_data.html Source data are provided with this paper.

## Source data

Source Data

## Code availability

Python code for data analysis is available under 10.5281/zenodo.7037530. This study uses JULES, two branches of JULES-vn5.2. https://code.metoffice.gov.uk/trac/jules/browser/main/branches/dev/linamercado/r14338_circadian at revision 22682 for TDQ_10_ simulations and https://code.metoffice.gov.uk/trac/jules/browser/main/branches/dev/douglasclark/vn5.2_diurnal_resp at revision 22681 for simulations with constant Q_10_ which are available on the Met Office Science Repository System (MOSRS; https://code.metoffice.gov.uk/trac/jules; registration required https://jules.jchmr.org/content/getting-started). Simulations were performed using Rose suites u-ce999 (new formulation) and u-ce859 for simulations with constant Q_10_, and u-bs101 (with new formulation) and u-ce767 for simulations with TDQ_10_ also available through MOSRS.
