# Supplementary material for: Nocturnal plant respiration is under strong non-temperature control
Source: Nat Commun. 2022 Sep 26;13:5650. doi: 10.1038/s41467-022-33370-1 (PMC9512894; doi:10.1038/s41467-022-33370-1)
Supplement: Supplementary file 1 — Supplementary Information [file 41467_2022_33370_MOESM1_ESM.pdf]

Supplementary Information

Supplementary Figure 1a

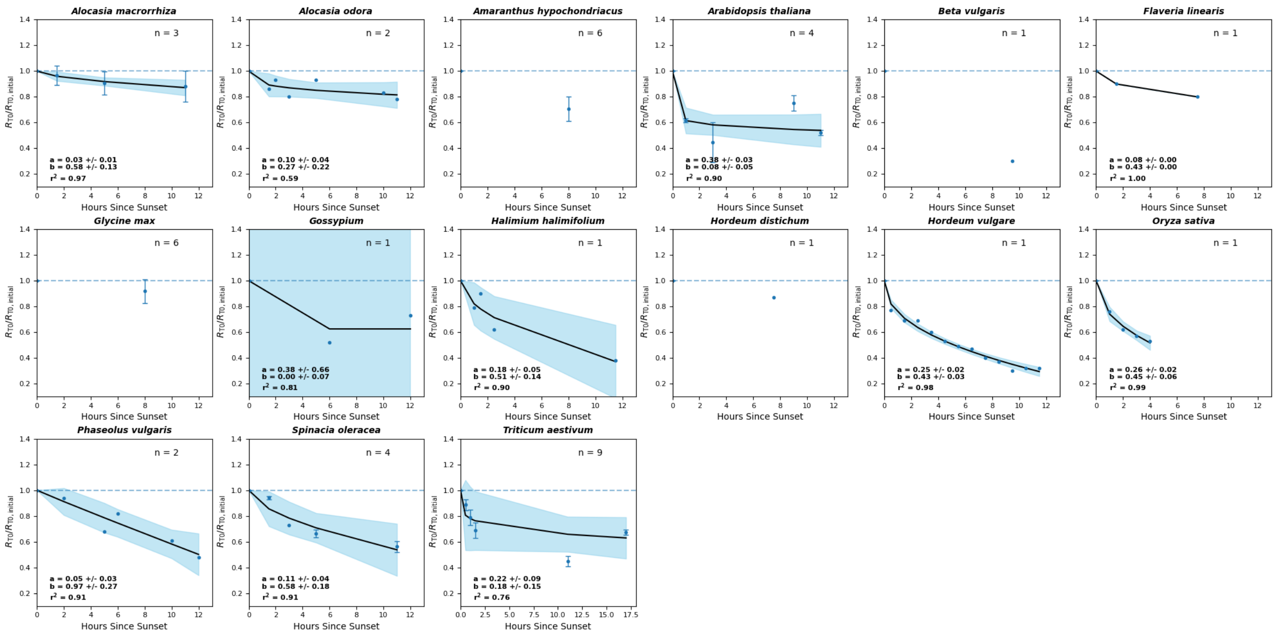

Supplementary Figure 1b

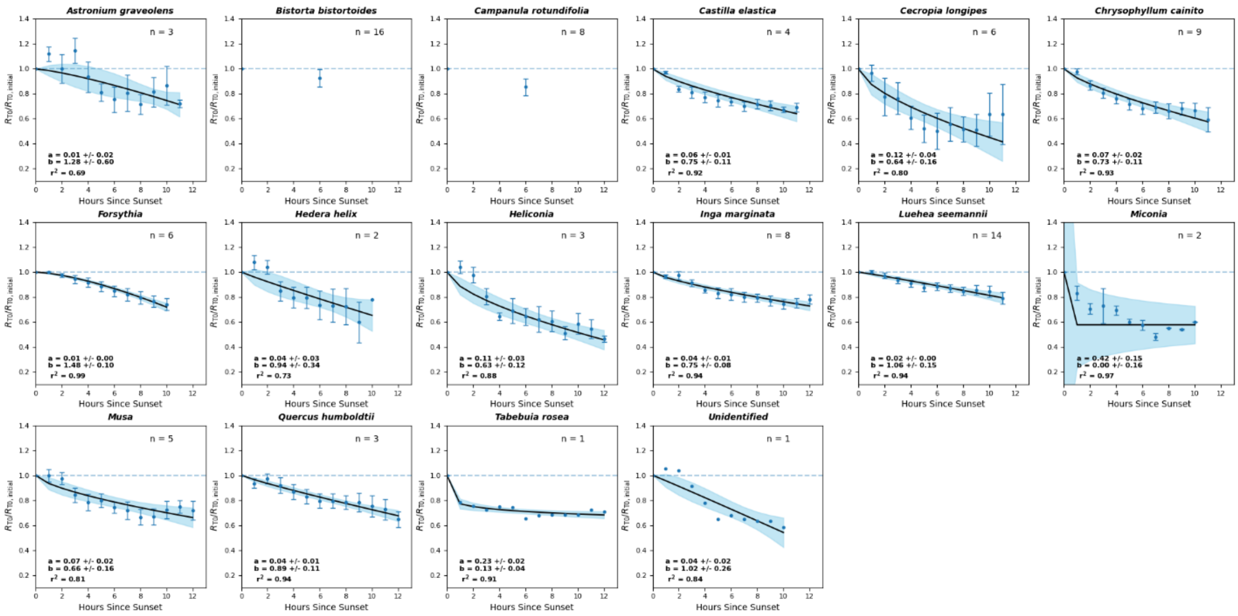

Supplementary Figure 1. (**a**, lab; **b**, field) Development of $R_{\mathrm{To}}/R_{\mathrm{To\text{-}initial}}$ (mean ± SEM) through the night from onset of darkness/sunset in individual species (Supplementary Table 1). Also shown are the 95%-confidence intervals in blue, estimates of the coefficients (including 1SD) of the power-functions ($y = 1 - a * \mathrm{hour}^{b}$), and the coefficient of determination (r^2). n denotes number of replicate plants per species. The results did not vary significantly (t-test) between biomes ($t = -1.116$, $df = 19.614$, p-value = 0.2779), experimental conditions ($t = 1.0819$, $df = 21.025$, p-value = 0.2915), or plant type ($t = -1.3837$, $df = 27.219$, p-value = 0.1777), allowing the entire dataset to be collated and a single universal equation to be derived for modelling, representative of all groups (Fig. 1a). Data are available in Supplementary Data 1.

Supplementary Figure 2a

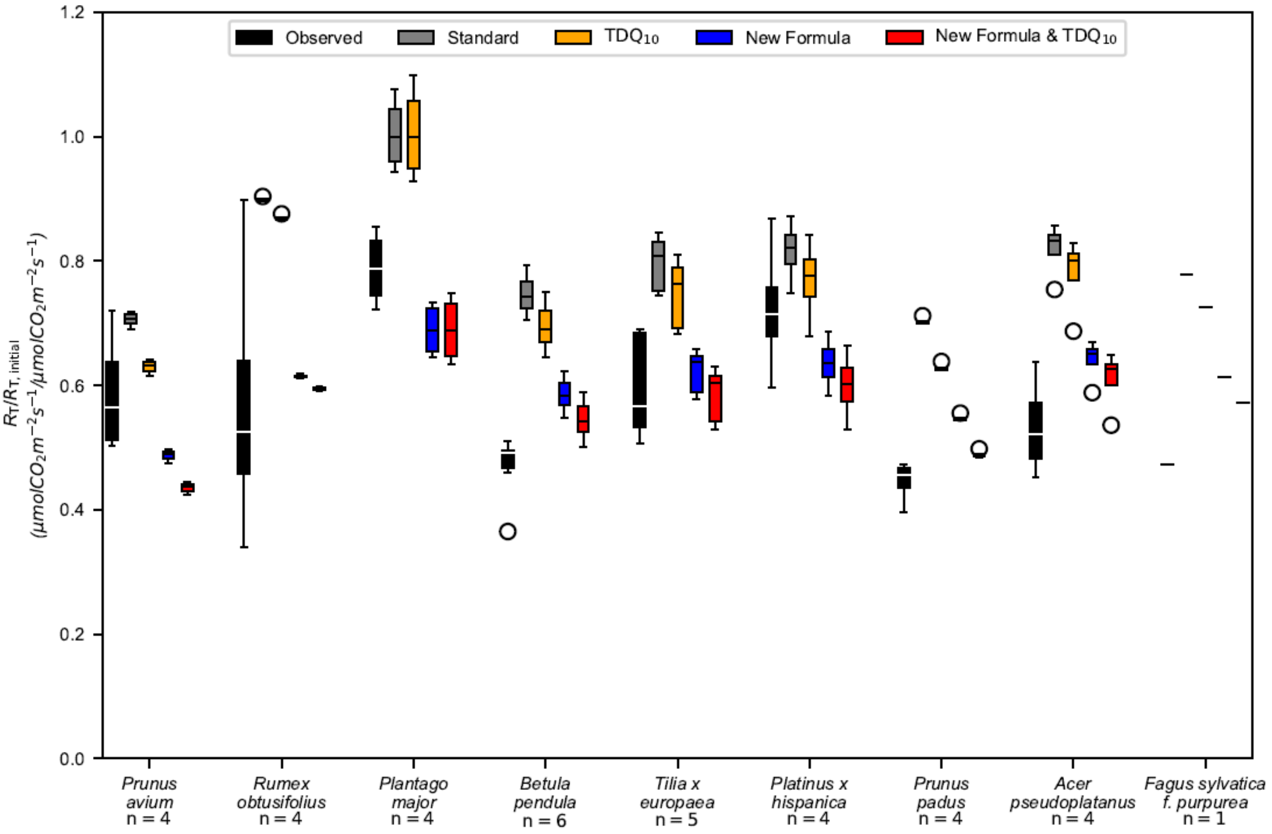

Supplementary Figure 2b

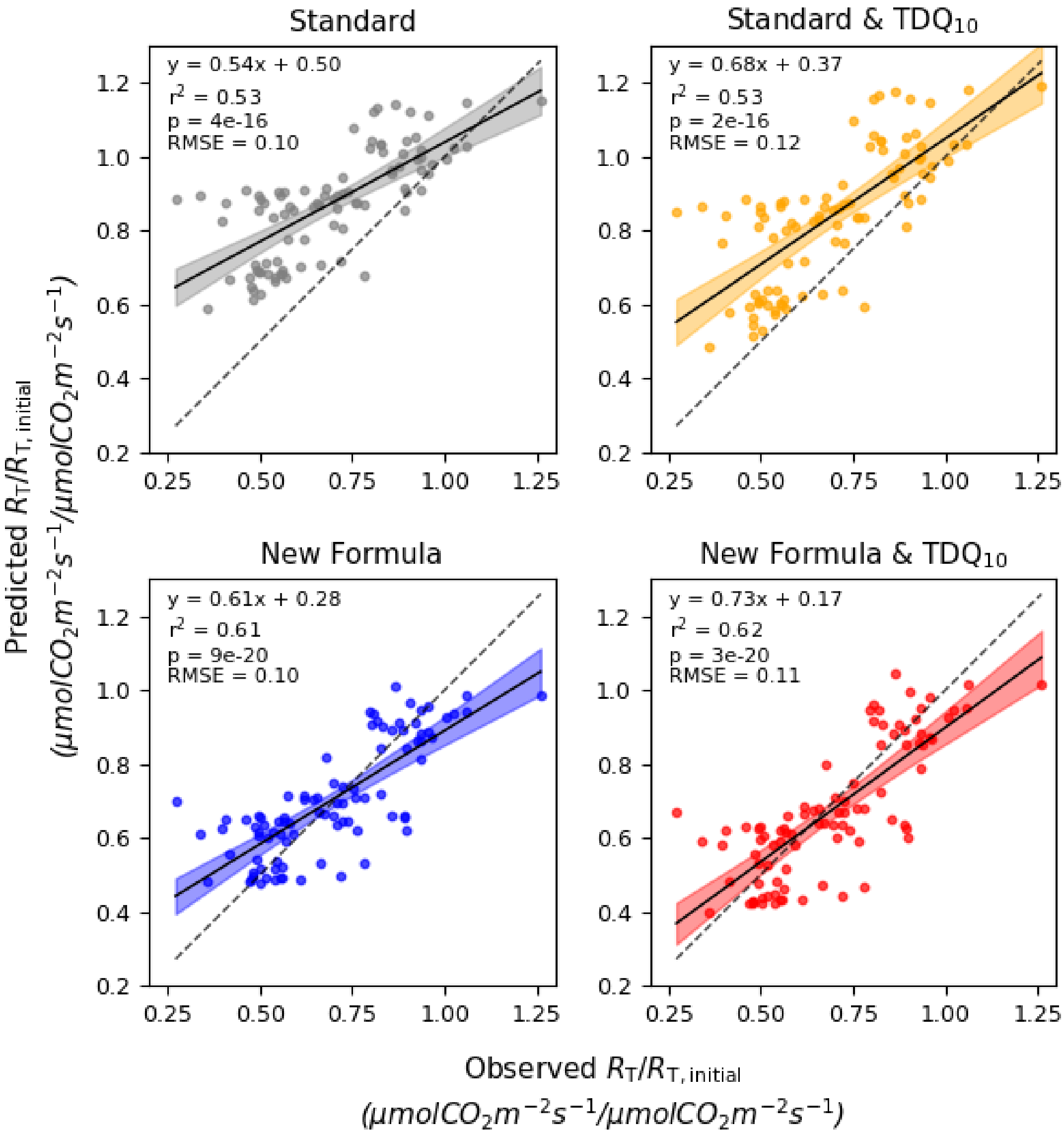

Supplementary Figure 2c

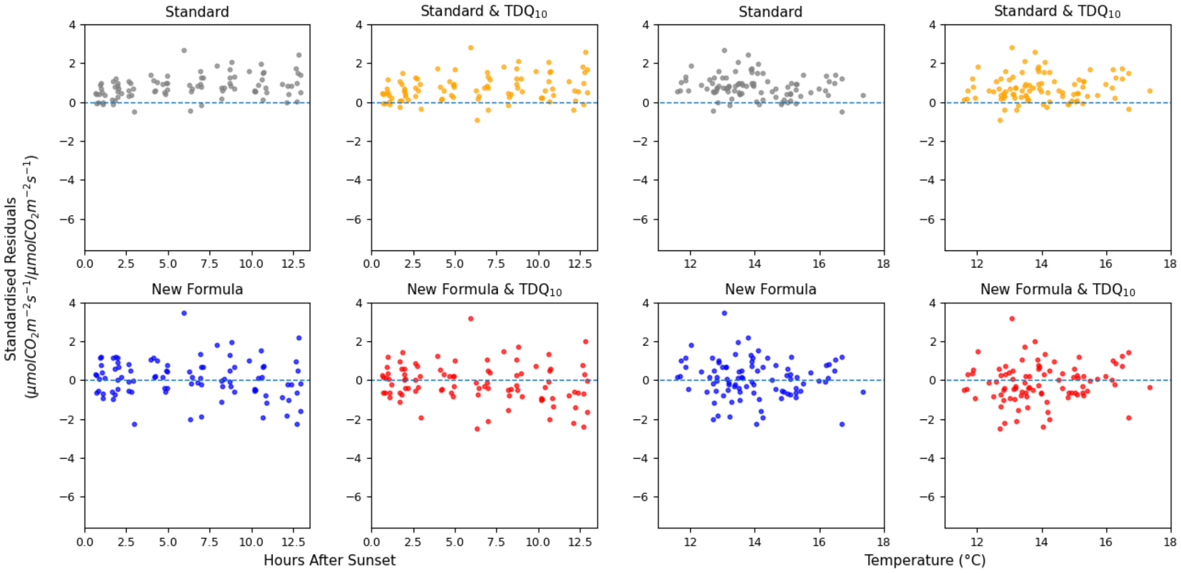

Supplementary Figure 2d

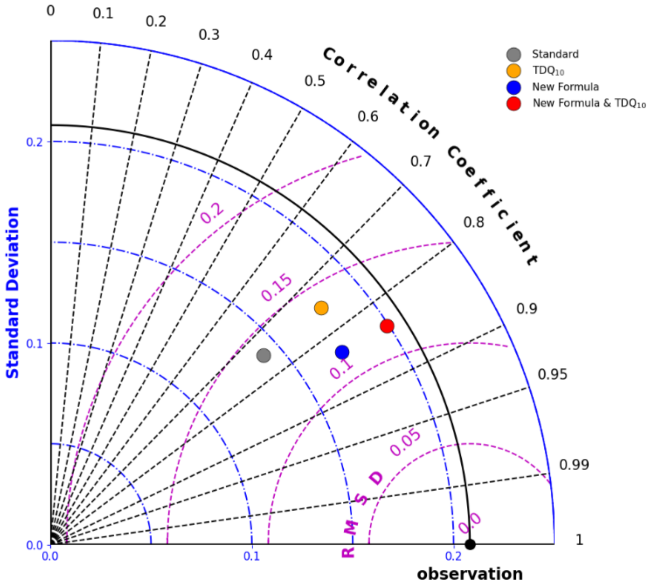

Supplementary Figure 2. A) Observed and modelled $R_{To}/R_{To\text{-initial}}$ in nine field-grown broad leaf species (Fig. 2c, Supplementary Table 3) at 13h after sunset. Modelled values are Standard (Equation 1 & Q_{10} = 2), Standard modified (Equation 1 & TDQ_{10}), New formulation (Equation 4 & Q_{10} = 2), and New formulation modified (Equation 4 & TDQ_{10}). **B)** Box-and-whisker-plots (The centre line is the median. The lower whisker is the lowest datum above the first quartile - 1.5*interquartile range. The upper whisker is the highest datum below the first quartile - 1.5*interquartile range. Any points outside the whiskers are plotted separately) of observed- and modelled leaf R_{To}/R_{To}-initial during nights for three species (Fig. 2c, n = 4 per species). **C)** Standardised residuals of the four simulations (S1-S4, Supplementary Table 4) over time after sunset and over air temperature. The residuals appear more symmetrically distributed for the models that include the new term including time of night. **D)** Model evaluation with a Taylor Diagram showing the models that include TDQ_{10} and Q_{10} = 2 and the new formula having better performance (highest correlation coefficient, closest standard deviation to observed and lowest RMSD) than models without the new formula. Data are available in Supplementary Data 2-3.

Supplementary Figure 3a

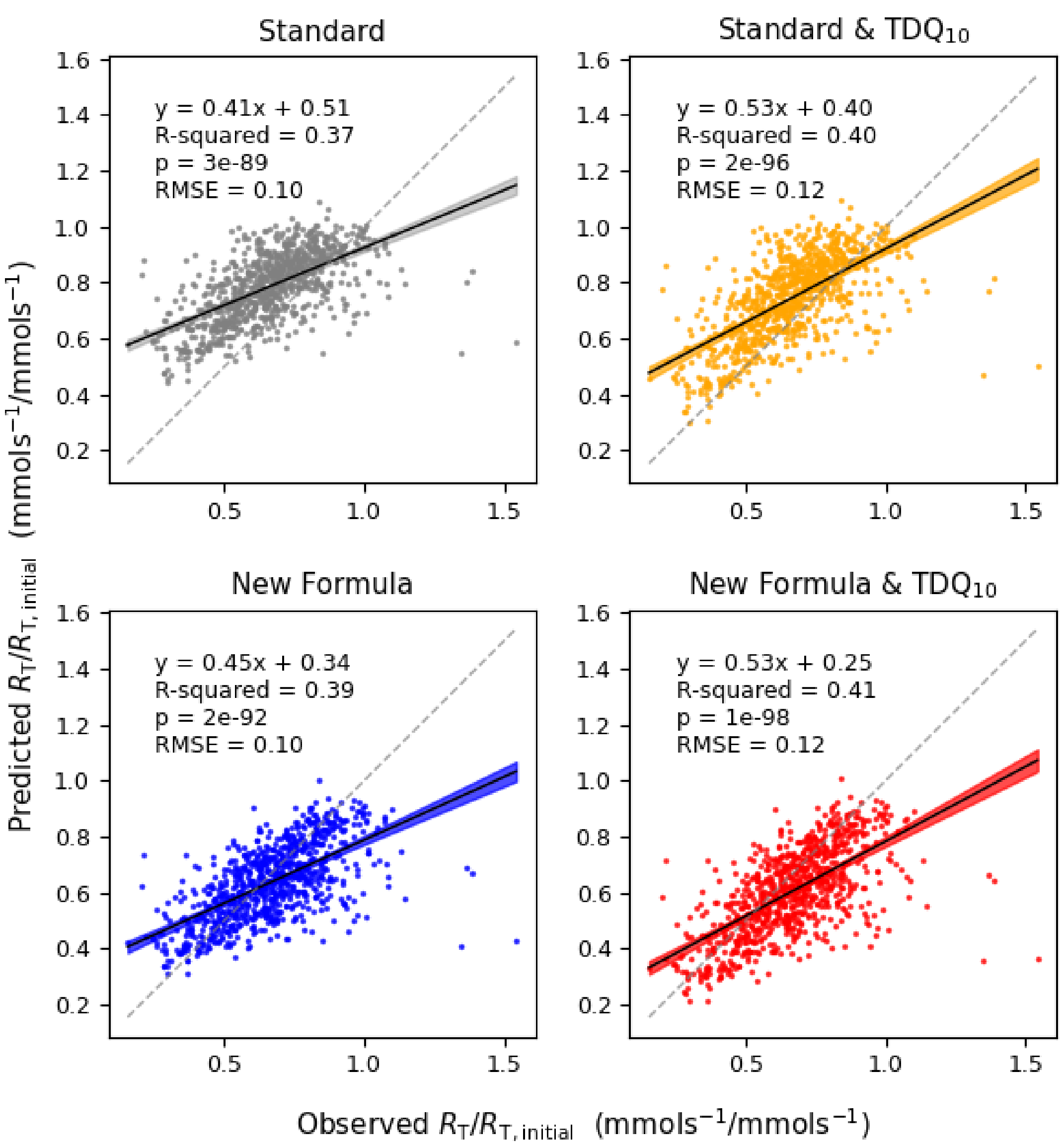

Supplementary Figure 3b

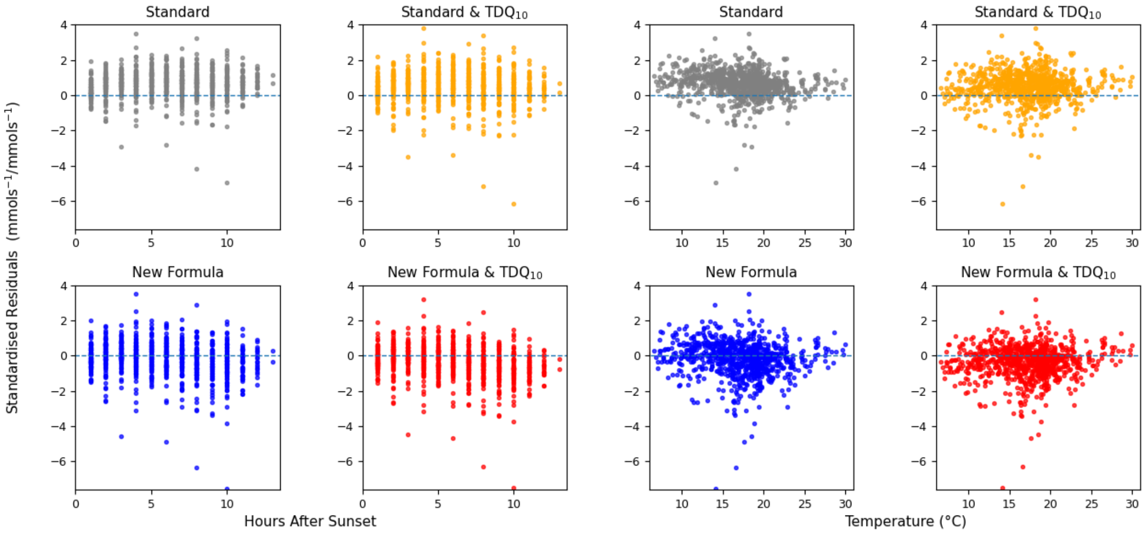

Supplementary Figure 3c

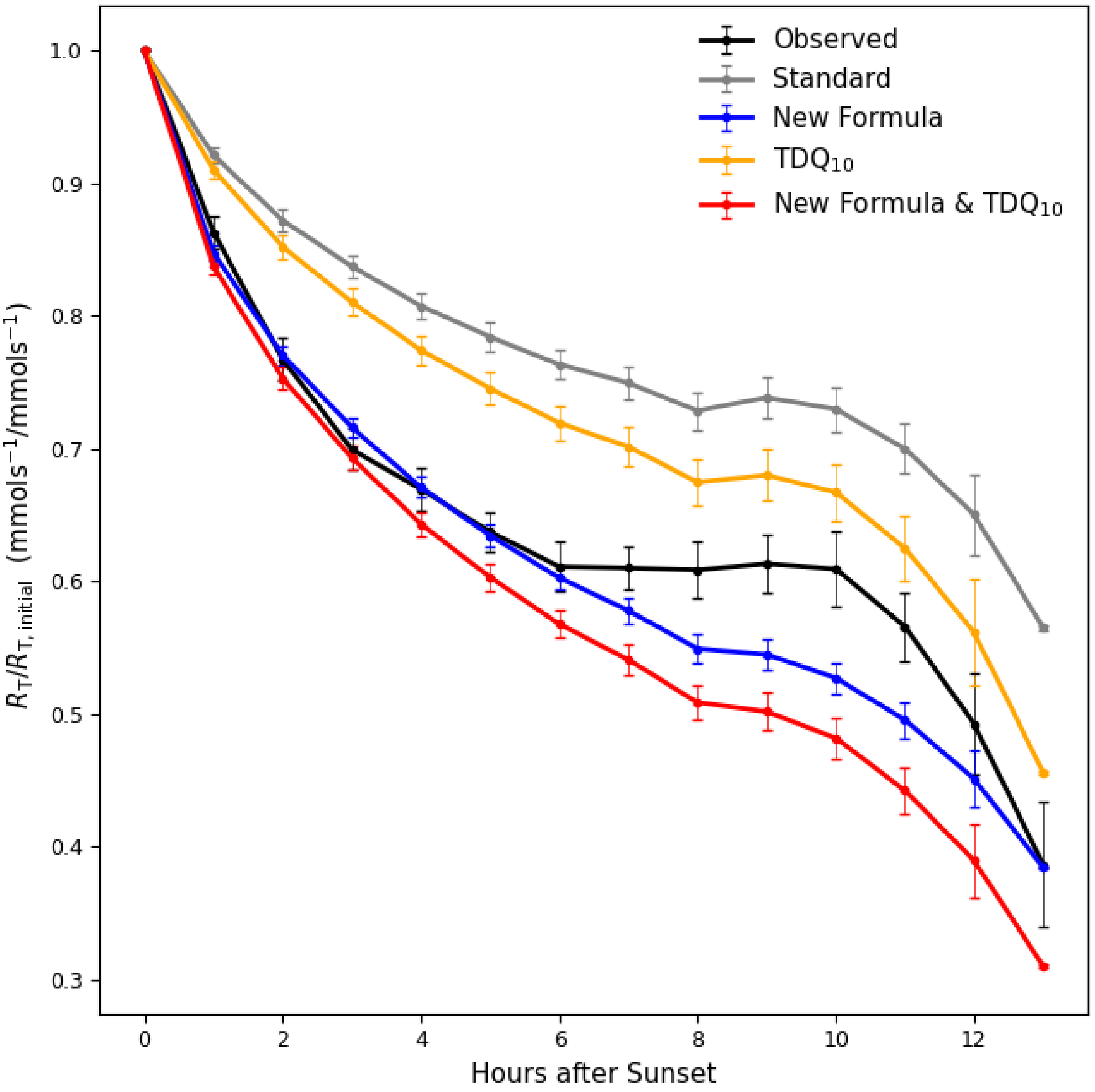

Supplementary Figure 3d

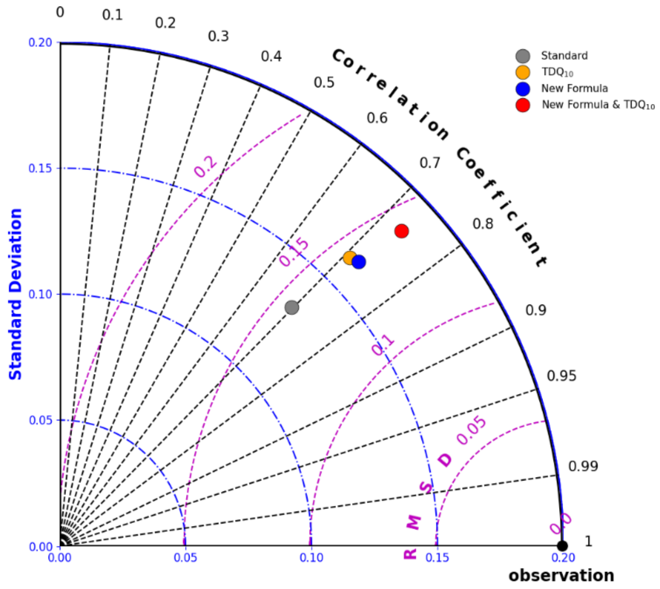

Supplementary Figure 3. Evaluation of Equation 4 using tree stand (*Eucalyptus tereticornis*) level measured- and modelled (S1-S4, Supplementary Table 4) values of $R_{\mathrm{To}}/R_{\mathrm{To\text{-}initial}}$. **A)** Predicted $R_{\mathrm{To}}/R_{\mathrm{To\text{-}initial}}$ as a function of measured $R_{\mathrm{To}}/R_{\mathrm{To\text{-}initial}}$ (replicate chambers = 3, number of nights = 62, period = 8- 13 hours), Root Mean Square Error (RMSE) are also given. **B)** Standardised residuals of the four simulations (S1-S4, Supplementary Table 4) over time after sunset and over air temperature. The residuals appear more symmetrically distributed for the models that include the new term including time of night. **C)** Measured- and modelled (S1-S4, Supplementary Table 4) values of $R_{\mathrm{To}}/R_{\mathrm{To\text{-}initial}}$ (replicate chambers = 3, number of nights = 62) plotted as function of time of night (means ± 1SD). **D)** Model evaluation with a Taylor Diagram showing the models that include TDQ_{10} and Q_{10} = 2 and the new formula having better performance (highest correlation coefficient, closest standard deviation to observed and lowest RMSD) than models without the new formula. Data are available in Supplementary Data 5.

Supplementary Figure 4

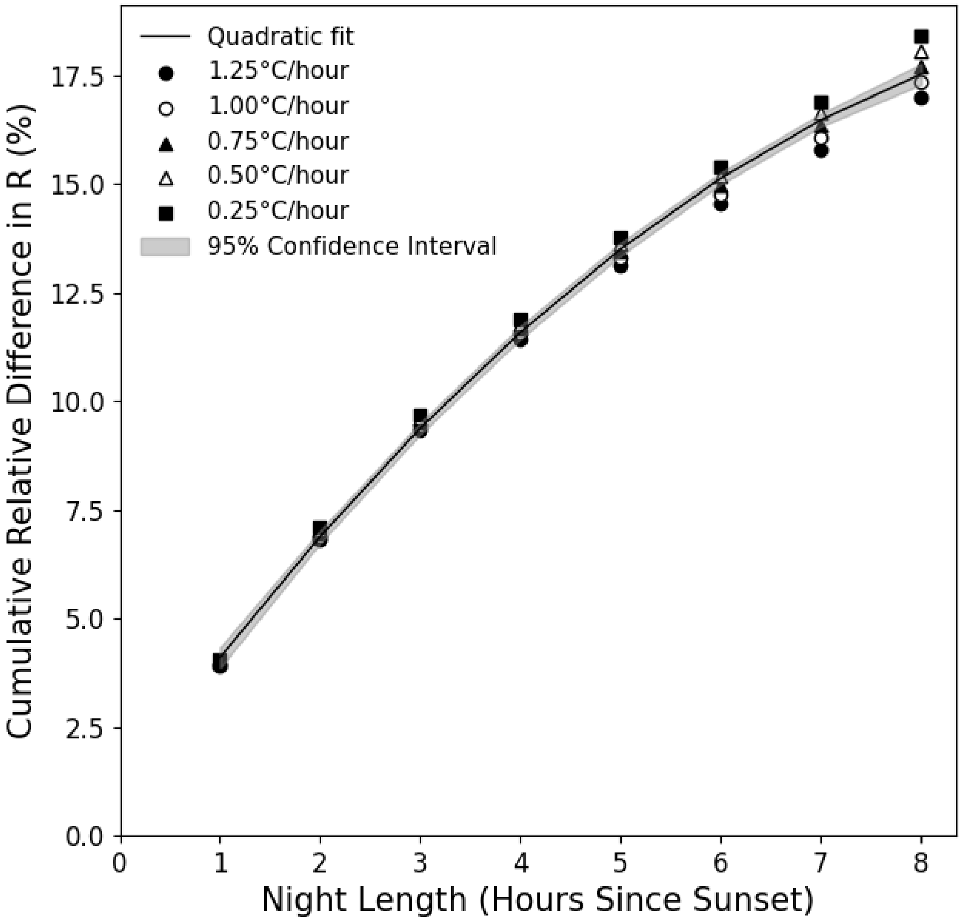

Supplementary Figure 4. Cumulated overestimation of R_T as function of length of night ($y = 1.0 + 3.2X - 0.14X^2$, quadratic fit, $R^2 = 0.995$, intercept $p = 6.45E\text{-}06$, X $p = 9.71E\text{-}29$, X^2 $p = 9.58E\text{-}16$) using Equation 1 instead of Equation 3 (mean response of five different rates of cooling at night). Data are available in Supplementary Data 6.

Supplementary Figure 5

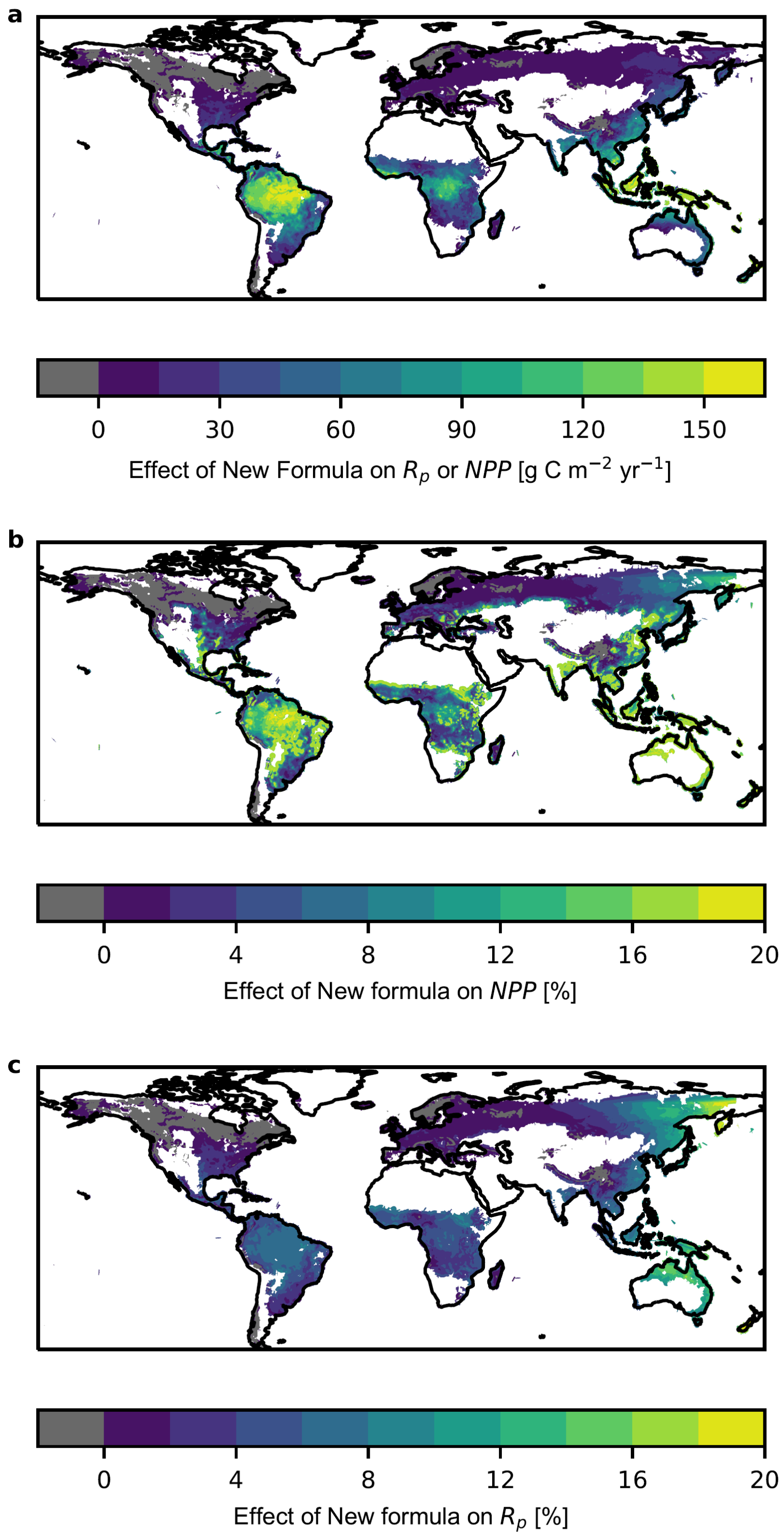

Supplementary Figure 5. Impact of incorporation of nocturnal variation in whole plant R_{To} in simulated reduction in plant respiration R_p (**A**, **C**) and corresponding increase in NPP (**B**) over the period 2000-2018 using TDQ$_{10}$ (eqn 2) and the new formula (eqn 5). Impact is estimated as the difference between the temporal mean of simulations with and without nocturnal variation in whole plant R_{To} for *NPP* and vice versa for R_p (**A**) and as a percentage respect to simulations without nocturnal variation in R_{To} (**B**, **C**). Note, the reduction in R_p (**A**) is identical to the increase in NPP in absolute terms. Results are presented for grid cells where grid level *NPP* is >50 g m^{-2} yr $^{-1}$ in the standard TDQ$_{10}$ simulations to avoid excessively large % effects at very low *NPP*.

Supplementary Table 1. Meta data underlying Figure 1a**.** Replicates within species indicate from published studies number of different values across different conditions that are possible to extract from the total number references for a species. Each value is typically based on several true replicates, a number that is not always possible to extract from the published studies.

**Species**	**Biome**	**Plant functional type**	**Experimental conditions**	**Woody or non-woody**	**Replicate**	**Reference**
Alocasia macrorrhiza	Tropical	Herbaceous	Lab	non-woody	3	Noguchi *et al.* 1996
Alocasia odora	Tropical	Herbaceous	Lab	non-woody	2	Noguchi & Terashima 1997; Noguchi *et al.* 2001
Amaranthus hypochondriacus	Tropical	Herbaceous	Lab	non-woody	6	Bunce 2007
Arabidopsis thaliana	Temperate	Herbaceous	Lab	non-woody	4	Trethewey & ap Rees 1994; Watanabe *et al.* 2014
Astronium graveolens	Tropical	Tree	Field	woody	3	This study
Beta vulgaris	Temperate	Herbaceous	Lab	non-woody	1	Fondy & Geiger 1982
Bistorta bistortoides	Temperate	Herbaceous	Field	non-woody	16	McCutchan & Monson 2001
Campanula rotundifolia	Temperate	Herbaceous	Field	non-woody	8	McCutchan & Monson 2001
Castilla elastica	Tropical	Tree	Field	woody	4	This study
Cecropia longipes	Tropical	Tree	Field	woody	6	This study
Chrysophyllum cainito	Tropical	Tree	Field	woody	9	This study

Flaveria linearis	Tropical	Herbaceous	Lab	non-woody	1	Leonardos et al 2006
Forsythia	Temperate	Shrub	Field	woody	6	This study
Glycine max	Tropical	Herbaceous	Lab	non-woody	6	Bunce 2007
Gossypium	Temperate	Shrub	Lab	woody	1	Gessler *et al.* 2017
Halimium halimifolium	Temperate	Herbaceous	Lab	non-woody	1	Lehmann *et al.* 2016
Hedera helix	Temperate	Vine	Field	woody	2	This study
Heliconia	Tropical	Herbaceous	Field	non-woody	3	This study
Hordeum distichum	Temperate	Herbaceous	Lab	non-woody	1	Farrar & Farrar 1985
Hordeum vulgare	Temperate	Herbaceous	Lab	non-woody	1	Baysdorfer *et al.* 1987
Inga marginata	Tropical	Tree	Field	woody	8	This study
Luehea seemannii	Tropical	Tree	Field	woody	14	This study
Miconia	Tropical	Herbaceous	Field	non-woody	2	This study
Musa	Tropical	Herbaceous	Field	non-woody	5	This study
Oryza sativa	Tropical	Grass	Lab	non-woody	1	Giuliani *et al.* 2019
Phaseolus vulgaris	Temperate	Herbaceous	Lab	non-woody	2	Gessler *et al.* 2017; Noguchi *et al.* 2001
Quercus humboldtii	Tropical	Tree	Field	woody	3	This study
Spinacia oleracea	Temperate	Herbaceous	Lab	non-woody	4	Noguchi & Terashima 1995, Noguchi *et al.* 1996
Tabebuia rosea	Tropical	Tree	Field	woody	1	This study
Triticum aestivum	Temperate	Herbaceous	Lab	non-woody	7	Averill & ap Rees 1994

Triticum aestivum	Temperate	Herbaceous	Lab	non-woody	10	Averill & ap Rees 1994
Triticum aestivum	Temperate	Herbaceous	Lab	non-woody	1	Azcon-Bieto & Osmon 1983
Triticum aestivum	Temperate	Herbaceous	Lab	non-woody	5	Azcon-Bieto & Osmon 1983
Unidentified	Tropical	Herbaceous	Field	non-woody	1	This study

Supplementary Table 2.

Species	Number of replicates	Growth conditions	Source	Inherent Q_{10}	Apparent Q_{10}	Temperature Control
Acer pseudoplatanus	1	Field	This study	1.8	3	0.57
Betula pendula	6	Field	This study	2	6.5	0.43
Eucalyptus pauciflora (autumn)	5	Field	Bruhn et al. 2007	1.7	4.2	(0.22)
Eucalyptus pauciflora (spring)	3	Field	Bruhn et al. 2008	2	2.8	(0.56)
Eucalyptus pauciflora (summer)	3	Field	Bruhn et al. 2008	2	2.7	(0.59)
Fagus sylvatica f. purpurea	1	Field	This study	2.6	7.9	0.32
Musa acuminata	5	Growth cabinet	This study	1.5	3.5	(0.20)
Platinus x hispanica	4	Field	This study	1.7	3	0.49
Pringlea antiscorbutica	4	Field	Bruhn et al. 2008	1.6	2	(0.60)
Prunus padus	4	Field	This study	2.4	5	0.65
Solanum lycopersicum	5	Growth cabinet	This study	2.1	4.3	(0.33)
Tilia x europaea	5	Field	This study	1.9	4.9	0.41
Mean				1.94	4.15	0.48
SD				0.09	0.5	0.05

Supplementary Table 2. Values of temperature sensitivity (Q_{10}) and temperature control (TC, see Fig. 2a-b) of nocturnal leaf respiration rate (µmol CO_2 m^{-2} s^{-1}) in different species from this study and from all published literature. Calculations of TC followed the method explained in Figure 2. Values of TC in brackets are not included in the Mean ± SD as they are alternatively calculated as Alternative TC = ($Q_{10,inh}$ -1)/($Q_{10,app}$ -1) because values of α and β (*sensu* Fig. 2) were not available in published

studies. In calculations of the Alternative TC the value 1 is subtracted from the Q_{10}-values because 1 represents the point where the respiration is not temperature-dependent. Thus, the Alternative TC is defined only below the temperature optimum of the respiration rate.

Supplementary Table 3

Additional 14 species used to evaluate Equation 4 (which is based on 31 species). S1-S4 are fully explained in Supplementary Table 4.

**Species**	**Method**	**Method**	**Level**
	$\mathbf{Q_{10,inh} < Q_{10,app}}$	**Temporal R_T vs S1-S4**	
Acer pseudoplatanus	X		Leaf
Betula pendula	X		Leaf
Eucalyptus pauciflora	X		Leaf
Fagus sylvatica f. purpurea	X		Leaf
Eucalyptus tereticornis		X	Entire tree
Musa acuminata	X		Leaf
Plantago major		X	Leaf
Platinus x hispanica	X		Leaf
Pringlea antiscorbutica	X		Leaf
Prunus padus	X		Leaf
Prunus avium		X	Leaf
Rumex obtusifolius		X	Leaf
Solanum lycopersicum	X		Leaf
Tilia x europaea	X		Leaf

Supplementary Table 4.

Modelling protocol

Simulation	Description	Equation
S1	Standard<br>Standard formula with Q_{10}=2	$R_T = R_{To} \times Q_{10}^{(T-To)/10}$ with Q_{10}=2, To=25ºC<br>(Equation 1)<br>R_{To}, is leaf R at 25ºC estimated for each plant functional type (PFT) as a fraction (parameter f_d) of V_{cmax} at 25ºC. Values of f_d, V_{cmax} and R_{To} for the 9 PFTs used in Jules simulations included in this study are reported in Supplementary Table 5.
S2	New formula<br>Standard including variable nocturnal R_{To}<br><br>As above using upper confidence intervals from Equation 3 derived in Fig. 1a<br><br>As above using lower confidence intervals from Equation 3 derived in Fig. 1a	$R_{T,t} = R_{T,sunset} \times Q_{10}^{0.1\times(T,t - Tsunset)} \times (1 - 0.08 \times h^{0.58})$<br>(Equation 1 & 3 = Equation 4)<br><br>$R_{T,t} = R_{T,sunset} \times Q_{10}^{0.1\times(T,t - Tsunset)} \times (1 - 0.0703 \times h^{0.562})$<br><br>$R_{T,t} = R_{T,sunset} \times Q_{10}^{0.1\times(T,t - Tsunset)} \times (1 - 0.093 \times h^{0.521})$

		$R_{T,sunset}$, corresponds to the value of leaf R_T at sunset time under sunset Temperature (Tsunset), estimated with Equation 1
S3	TDQ_{10}<br>Standard with temperature dependent Q_{10} (TDQ_{10})	$R_T = R_{To} \times Q_{10}^{(T-To)/10}$ with Q_{10} = 3.09 - 0.0435*T<br>(Equation 1 & 2)
S4	New formula & TDQ_{10}<br>Standard including variable nocturnal R_{To} and TDQ_{10}<br><br>As above using upper confidence intervals from Equation 3 derived in Fig. 1a<br><br>As above using lower confidence intervals from Equation 3 derived in Fig. 1a	$R_{T,t} = R_{T,sunset} \times Q_{10}^{0.1\times(T,t - Tsunset)} \times (1 - 0.08 \times h^{0.59})$<br>With Q_{10} = 3.09 - 0.0435*T<br>(Equation 4 & 2 = Equation 5)<br><br>$R_{T,t} = R_{T,sunset} \times Q_{10}^{0.1\times(T,t - Tsunset)} \times (1 - 0.0703 \times h^{0.562})$<br>With Q_{10} = 3.09 - 0.0435*T<br><br>$R_{T,t} = R_{T,sunset} \times Q_{10}^{0.1\times(T,t - Tsunset)} \times (1 - 0.093 \times h^{0.521})$<br>With Q_{10} = 3.09 - 0.0435*T

Supplementary Table 5

R_{To} used in Jules simulations estimated as $R_{To} = f_d$ x V_{cmax} at 25°C used in Equations 1 &2, reported in Table 2 of reference 33.

Plant functional type	R_{To} [μmol m^2 s^{-1}]	f_d	V_{cmax} at 25°C [μmol m^2 s^{-1}]
Tropical broadleaf evergreen tree	0.41	0.01	41.16
Temperate broadleaf evergreen tree	0.61	0.01	61.28
Temperature broad leaf deciduous tree	0.57	0.01	57.25
Needle leaf evergreen tree	0.8	0.015	53.55
Needle leaf deciduous tree	0.76	0.015	50.83
C_3 grass	0.97	0.019	51.09
C_4 grass	0.6	0.019	31.71
Evergreen shrub	0.94	0.015	62.41
Deciduous shrub	0.76	0.015	50.40

Supplementary Table 6

Impact of incorporation of nocturnal variation in whole plant R_{To} in simulated plant respiration R_p and *NPP* in simulations with standard Q_{10}=2 and with TDQ_{10} using mean values, upper and lower confidence intervals (CI) (See equations in Supplementary Table 4). Impact is estimated in percentage as the difference between the temporal mean of simulations with and without nocturnal variation in whole plant R_{To} for *NPP* and vice versa for R_p divided by respective simulations without nocturnal variation in R_{To}. Calculations only include grid cells where grid level *NPP* is >50 g m^{-2} yr^{-1} in the respective standard simulations to avoid excessively large % effects at very low *NPP*.

Simulation	Global *NPP*	Global R_p	Tropical *NPP*	Tropical R_p
Standard Q_{10} mean values	8.8	5.0	10.2	5.2
Standard Q_{10} upper CI	8.0	4.5	9.2	4.7
Standard Q_{10} lower CI	10.0	5.7	11.5	5.9
TDQ_{10} mean values	7.9	5.2	10.2	5.9
TDQ_{10} upper CI	7.2	4.8	9.4	5.4
TDQ_{10} lower CI	8.8	5.9	11.4	6.6

Supplementary Table 7

Explanation of symbols and acronyms regarding respiration and its temperature-sensitivity

Acronym	Explanation
Q_{10}	T-sensitivity, is the relative change in *R* obtained with a 10°C change in T
$Q_{10,inh}$	Inherent Q_{10}: measured via short term (max 30 min) artificial T-changes
$Q_{10,app}$	Apparent Q_{10}: measured via longer (hours) term natural changes in T in the environment
R	Rate of respiration
R_{T}	*R* at any given T
R_{To}	*R* at set T, constant T
$R_{To\text{-}initial}$	Initial measurement of R_{To}
$R_{T,sunset}$	Rate of respiration at sunset in terms of time and temperature (Equation 5)
$R_{T,t}$	Rate of respiration at given timestep, t (Equations 4 & 5)
T	Temperature
T_{o}	Set temperature, constant temperature
T,t	Leaf Temperature at a given timestep (Equations 4 & 5)
T_{sunset}	Temperature at sunset
TC	Degree to which T (via $Q_{10,inh}$) determines temporal variation in *R*
TDQ_{10}	T-dependent Q_{10}

Supplementary References

1. Noguchi, K., Sonoike, K., Terashima, I. Acclimation of Respiratory Properties of Leaves of Spinacia oleracea L., a Sun Species, and of Alocasia macrorrhiza (L.) G. Don., a Shade Species, to Changes in Growth Irradiance. *Plant Cell Physiol.* **37**, 377-384 (1996).
2. Noguchi, K., Nakaijma, N., Terashima, I. Acclimation of leaf respiratory properties in *Alocasia odora* following reciprocal transfers of plants between high- and low-light environments. *Plant Cell Environ.* **24**, 831–839 (2001).
3. Bunce, J.A. Direct and Acclimatory Responses of Dark Respiration and Translocation to Temperature. *Ann. Bot.* **100**, 67–73 (2007).
4. Trethewey, R. N., ap Rees, T. The role of the hexose transporter in the chloroplasts of *Arabidopsis thaliana* L. *Planta* **195**, 168-174 (1994).
5. Watanabe, C. K., Sato, S., Yanagisawa, S., Uesono, Y., Terashima, I., Noguchi, K. Effects of Elevated CO_2 on Levels of Primary Metabolites and Transcripts of Genes Encoding Respiratory Enzymes and Their Diurnal Patterns in *Arabidopsis thaliana*: Possible Relationships with Respiratory Rates. *Plant Cell Physiol.* **55**, 341–357 (2014).
6. McCutchan, C. L., Monson, R. K. Night-time respiration rate and leaf carbohydrate concentrations are not coupled in two alpine perennial species. *New Phytol.* **49**, 419–430 (2001).
7. Leonardos, E. D., Micallef, B. J., Micallef, M. C., Grodzinski, B. Diel patterns of leaf C export and of main shoot growth for *Flaveria linearis* with altered leaf sucrose–starch partitioning. *J. Exp. Bot.* **57**, 801–814 (2006).
8. Lehmann, M. M., Wegener, F., Werner, R. A., Werner, C. Diel variations in carbon isotopic composition and concentration of organic acids and their impact on plant dark respiration in different species. *Plant Biol.* **18**, 776–784 (2016).

9. Farrar, S. C., Farrar, J. F. Carbon fluxes in leaf blades of Barley. *New Phytol*. **100**, 271-283 (1985).

10. Baysdorfer, C., Sicher, R. C., Kremer, D. F. Relationship between Fructose 2,6-Bisphosphate and Carbohydrate Metabolism in Darkened Barley Primary Leaves. *Plant Physiol*. **84**, 766-769 (1987).

11. Giuliani, R., Karki, S., Covshoff, S., Lin, H., Coe, R., Koteyeva, N., Quick, W., Von Caemmerer, S., Furbank, R., Hibberd, J., Edwards, G. and Cousins, A. Knockdown of glycine decarboxylase complex alters photorespiratory carbon isotope fractionation in *Oryza sativa* leaves. J. Exp. Bot. **70**, 2773-278. (2019).

12. Averill, R. H., ap Rees, T. The control of respiration in wheat (*Triticum aestivum* L.) leaves. *Planta* **196**, 344-349 (1995).

13. Azcón-Bieto, J., Relationship between Photosynthesis and Respiration: The Effect of Carbohydrate Status on the Rate of CO2 Production by Respiration in Darkened and Illuminated Wheat Leaves, *Plant Physiol*. **71**, 574-581 (1983).

14. Bruhn, D., Edgerton, J., Loveys, B. and Ball, M. Evergreen leaf respiration acclimates to long-term nocturnal warming under field conditions. *Glob. Change Biol.***13**, 1216-1223 (2007).
