# Supplementary material for: Nocturnal plant respiration is under strong non-temperature control
Source: Nat Commun. 2022 Sep 26;13:5650. doi: 10.1038/s41467-022-33370-1 (PMC9512894; doi:10.1038/s41467-022-33370-1)
Supplement: Supplementary file 2 — Description of Additional Supplementary Files [file 41467_2022_33370_MOESM2_ESM.pdf]

Description of Additional Supplementary Files

File Name: Supplementary Data 1
Description: Leaf $R_{\mathrm{To}}/R_{\mathrm{To,initial}}$ values of leaves through night-time for all species in Figure 1a and Supplementary Figure 1.

File Name: Supplementary Data 2
Description: Replicate values of observed and modelled $R_{\mathrm{T}}/R_{\mathrm{T\text{-}initial}}$ in nine field-grown broad leaf species in Figure 1b, 1c and Supplementary Figure 2 – Denmark

File Name: Supplementary Data 3
Description: Replicate values of observed and modelled $R_{\mathrm{T}}/R_{\mathrm{T\text{-}initial}}$ in nine field-grown broad leaf species in Figure 1b and Supplementary Figure 2 – Exeter

File Name: Supplementary Data 4
Description: Data for conceptual figure of how R_{T} (rate of respiration at varying temperature) measured in response to either i) common short-term rapid artificial cooling (e.g. during 10-30 min) of leaf/plant can be used to calculate $Q_{10,\mathrm{inh}}$ (inherent temperature sensitivity of *R*, Equation 1, using point connected by the grey dashed line), or ii) natural cooling (over several hours) of the environment along temporal variation in T can be used to calculate $Q_{10,\mathrm{app}}$ (apparent temperature sensitivity of *R*). Data of $Q_{10,\mathrm{app}}$, $Q_{10,\mathrm{inh}}$ and TC (temperature control) of *R* ($\alpha/(\alpha+\beta)$) across ten species (46 replicate plants). Data of the dependence of Q_{10} and TC of *R* on rate of nocturnal cooling. Data of modelled nocturnal variations in *R* in response to T decrease during the night including and excluding effects of non-temperature control on metabolism. Figure 2.

File Name: Supplementary Data 5
Description: Data for evaluation of Equation 4 using tree stand (*Eucalyptus tereticornis*) level measured- and modelled (S1-S4, Supplementary Table 4) values of $R_{\mathrm{To}}/R_{\mathrm{To\text{-}initial}}$. **A)** Predicted $R_{\mathrm{To}}/R_{\mathrm{To\text{-}initial}}$ as a function of measured $R_{\mathrm{To}}/R_{\mathrm{To\text{-}initial}}$ (replicate chambers = 3, number of nights = 62, period = 8- 13 hours), Root Mean Square Error (RMSE) are also given. **B)** Standardised residuals of the four simulations (S1-S4, Supplementary Table 4) over time after sunset and over air temperature. The residuals appear more symmetrically distributed for the models that include the new term including time of night. **C)** Measured- and modelled (S1-S4, Supplementary Table 4) values of $R_{\mathrm{To}}/R_{\mathrm{To\text{-}initial}}$ (replicate chambers = 3, number of nights = 62) plotted as function of time of night (means ± 1SD). **D)** Model evaluation with a Taylor Diagram showing the models that include TDQ_{10} and $Q_{10} = 2$ and the new formula. Supplementary Figure 3.

File Name: Supplementary Software
Description: zip folder with the code (one python file for plotting figures, another which modifies a library, as explained in the readme). Data for Supplementary Figure 4 is included in this code.
