# Supplementary material for: Nocturnal plant respiration is under strong non-temperature control
Source: Nat Commun. 2022 Sep 26;13:5650. doi: 10.1038/s41467-022-33370-1 (PMC9512894; doi:10.1038/s41467-022-33370-1)
Supplement: Supplementary file 8 — Reporting Summary [file 41467_2022_33370_MOESM8_ESM.pdf]

nature portfolio

Corresponding author(s): Dan Bruhn, Lina M Mercado

Last updated by author(s): Aug 25, 2022

Reporting Summary

Nature Portfolio wishes to improve the reproducibility of the work that we publish. This form provides structure for consistency and transparency in reporting. For further information on Nature Portfolio policies, see our Editorial Policies and the Editorial Policy Checklist.

Statistics

For all statistical analyses, confirm that the following items are present in the figure legend, table legend, main text, or Methods section.

n/a	Confirmed	
[ ]	[x]	The exact sample size (*n*) for each experimental group/condition, given as a discrete number and unit of measurement
[ ]	[x]	A statement on whether measurements were taken from distinct samples or whether the same sample was measured repeatedly
[ ]	[x]	The statistical test(s) used AND whether they are one- or two-sided<br>*Only common tests should be described solely by name; describe more complex techniques in the Methods section.*
[x]	[ ]	A description of all covariates tested
[x]	[ ]	A description of any assumptions or corrections, such as tests of normality and adjustment for multiple comparisons
[ ]	[x]	A full description of the statistical parameters including central tendency (e.g. means) or other basic estimates (e.g. regression coefficient) AND variation (e.g. standard deviation) or associated estimates of uncertainty (e.g. confidence intervals)
[ ]	[x]	For null hypothesis testing, the test statistic (e.g. *F*, *t*, *r*) with confidence intervals, effect sizes, degrees of freedom and *P* value noted<br>*Give P values as exact values whenever suitable.*
[x]	[ ]	For Bayesian analysis, information on the choice of priors and Markov chain Monte Carlo settings
[x]	[ ]	For hierarchical and complex designs, identification of the appropriate level for tests and full reporting of outcomes
[x]	[ ]	Estimates of effect sizes (e.g. Cohen's *d*, Pearson's *r*), indicating how they were calculated

Our web collection on statistics for biologists contains articles on many of the points above.

Software and code

Policy information about availability of computer code

Data collection	Jules model source code (JULES-vn5.2) and Rose suites to run the model are available via the Met Office Science Repository Service.
Data analysis	All data analysis including statistical analysis and figures of RTo were performed using Python version 3.9.4. Model evaluation, statistical analysis and figures were done using python version 3.9.4. Output from JULES was analysed and plotted using python version 2.7.16.

For manuscripts utilizing custom algorithms or software that are central to the research but not yet described in published literature, software must be made available to editors and reviewers. We strongly encourage code deposition in a community repository (e.g. GitHub). See the Nature Portfolio guidelines for submitting code & software for further information.

Data

Policy information about availability of data

All manuscripts must include a data availability statement. This statement should provide the following information, where applicable:

- Accession codes, unique identifiers, or web links for publicly available datasets
- A description of any restrictions on data availability
- For clinical datasets or third party data, please ensure that the statement adheres to our policy

Data are available in Supplementary Materials. Jules model output available upon request during the review process and will be archived in an open access server before publication

Field-specific reporting

Please select the one below that is the best fit for your research. If you are not sure, read the appropriate sections before making your selection.

☐ Life sciences ☐ Behavioural & social sciences ☒ Ecological, evolutionary & environmental sciences

For a reference copy of the document with all sections, see nature.com/documents/nr-reporting-summary-flat.pdf

Ecological, evolutionary & environmental sciences study design

All studies must disclose on these points even when the disclosure is negative.

Study description	Data were obtained from i) published material and own ii) lab + field studies. For ii) a minimum of four replicate plants per species were measured throughout night-time. Two factors were considered in the study: species and time of night. However, as all measurements were not conducted at the same time, due to practical reasons, it is not a true factorial design.
Research sample	Taxa is higher plants. Samples represent primarily herbaceous and tree species. Literature values were obtained via search for articles published on leaf respiration in Web of Science. Thus, literature data were not from a data base per se.
Sampling strategy	Sampling size was chosen to ensure a minimum of four replicates in the field per species. The reasonings for this choice were i) ability to calculate a variation around the mean for each time point through the night, ii) time course though the night provides power for the analyses of time effect (Fig1), and iii) we prioritised to sample as many species as possible to be able to see a general trend for plants
Data collection	DB retrieved literature data. Data were collected at night-time by the authors. DB, FN, PP, MS, SS, JD, MGT, SS, and LMM conducted new field and lab measurements. leaf CO2 exchange was measured with with infra-red gas analysers (LiCor-6400(XT) or LiCor-6800, Lincoln, Nebraska, USA) in field and lab, whereas an automated system (LiCor-7000, Lincoln, Nebraska, USA) measured the net exchange of CO2 between the canopy and the atmosphere within each chamber at 15-min resolution.
Timing and spatial scale	Data were samples between ever 3 minutes and 1 hour though the night-time in order to sufficiently describe the temporal resolution of respiration. Replicate plants within a species were within a maximum of 50 m. Dates for measurements were based on when field campaigns were practically and logistically possible. Due to limited number of portable gas analyses, there were gaps between species. Measurement dates for own samples - DK: Prunus avium 23/9-2020, Rumex obtusifolius - 27/9-2020, Plantago major - 29/9-2020. UK: Betula pendula: July 4th-5th & July 5th-6th 2017, Tilia x europaea: July 6th-7th & 13th-14th, Platinus x hispanica: July 12th-13th & July 13th-14th, Prunus padus: July 16th-17th, Acer pseudoplatanus: July 12th-13th & 25th-26th Fagus sylvatica f. purpurea: July 5th-6th 2017, Forsythia August 1-7 2017. Panama: Luehea seemannii 11/10 2012 - 17/11 2012, Castilla elastica 9-14/10 2012, Astronium graveolens 2-22/7 2012, Cecropia longipes 22/6-1/7 2012, Chrysophyllum cainito 14-21/6 2012. Brazil: Heliconia 2-4/10 2018, Miconia 4-6/10 2018, Musa 24-26/10 2018. Columbia: Inga marginata 13-20/7 2019, Quercus humboldtii 8-21/7 2019, Tabebuia rosea 28-30/7 2019. Australia: Eucalyptus tereticornis 4/12/2013 to 25/5/2014.
Data exclusions	For the chamber measurements: Due to high noise-to-signal ratio in the CO2-exchange measurements from this system when analysing the high-resolution temporal variation through each night, we chose to only analyse temporal variation in tree-RT for the nights when tree-RT-initial were amongst the top 10% of CO2-exchange signals for the entire data set. The resulting data spanned 62 nights and included hourly average measurements from three replicate chambers.
Reproducibility	Data were obtained from publish literature and own lab+field measurements (31 species) and conclusions were confirmed in another 14 species by own measurements
Randomization	Random sampling was applied
Blinding	Blinding was not applied as entire nights were sampled. Thus, the person conduction set measurements were not affecting the automated measuremets

Did the study involve field work? ☒ Yes ☐ No

Field work, collection and transport

Field conditions	Denmark: air temperature ranged from 8.4 - 19.4 °C during measurement period, no rainfall during measurement period. UK: July: air temperature ranged from 13.93 - 23.75 °C, rainfall 36.3 mm; August: air temperature ranged from 14.08 - 23.31 °C, rainfall 53.02 mm. Panama: Mean annual temperature is 26.9◦C with mean daily maximum of 32.6C. Mean annual rainfall ~2260 mm, 80% of which falls in the May-Dec wet season. Brazil: Mean air temperature is 26°C and annual precipitation ranges from 1,900 – 2,500 mm with a pronounced dry season from June to October. Columbia: mean annual temperatures of 14, 22 and 26°C and mean annual precipitation of 2774, 2045 and 2298 mm/year.
Location	Northern Jutland, Denmark<br>Gamboa, Republic of Panamá.<br>Caramanta, Tamesis and Puente Iglesias in Antioquia, Colombia,<br>Manaus, Brazil,<br>Exeter and London, UK

	Measurement in Denmark taken Measurements of Rdark in the field were conducted on three wild danish species; Prunus avium (L.), Rumex obtusifolius (L.) and Plantago major (L.), each with four specimens. Each of the specimens were selected in approximately the same area, to approach near identical conditions in terms of light, water and nutrient availability. Furthermore, leaves from P. avium were selected among the lower canopy. The field studies were conducted in September 2020 and locations of the three species in the field were as follows: P. avium (57°01'00.9"N 9°59'16.9"E), R. obtusifolius and P. major (57°15'08.0"N 10°12'43.4"E), all in northern Jutland, Denmark. P. avium, located in a hedgerow. R. obtusifolius and P. major, located in a private garden. Bergenia cordifolia 'rotblum' were cultivated potted plants.<br><br>The measurements from Panama were taken in Gamboa, Colón Province. Gamboa is on the edge of Soberanía National Park. The plants that were measured here were not in the national park, but in a residential garden <200 m from the forest edge. Mean annual temperature is 26.9◦C with mean daily maximum of 32.6C. Mean annual rainfall ~2260 mm, 80% of which falls in the May-Dec wet season.<br><br>Measurements in Brazil were taken at a field station in Central Amazonia (2° 30`S, 60° W) 80 km north of Manaus city during the dry season months. Data was collected on attached leaves from trees within the field station.<br><br>Measurements in Colombia were taken on attached leaves from 2.5 year old juvenile trees located in private land at three locations (5.54°N, -75.68°W, 5.61°N, -75.62°W, 6.01°N -75.85° W) on an elevation gradient (2452, 1326, 575 masl) in the Colombian Andes with correspondent mean annual temperatures of 14, 22 and 26°C and mean annual precipitation of 2774, 2045 and 2298 mm/year.<br><br>Measurements in the UK were at done on attach leaves from trees at the University of Exeter campus and on a private garden in London during summertime months.
Access & import/export	No permit was required in Denmark as measurements were taken in private land (of author) and public land.<br><br>Data were collected under Panama department of the Environment (current name MiAmibiente) research permit under the name of Dr Kaoru Kitajima. Permit number: SE/P-16-12.<br><br>Data in Brazil were collected under the minister of Environment (Ministério do Meio Ambiente - MMA), Instituto Chico Mendes de Conservação da Biodiversidade - ICMBio, Sistema de Autorização e Informação em Biodiversidade - SISBIO permit number 47080-3.<br><br>No permit was required in Colombia as measurements were taken in private land, no plant samples were collected and trees were part of an existing experiment for which one of the coauthors is the lead.<br><br>No access permits were required in the UK as they were conducted on campus of own university + in own private garden.
Disturbance	Collection of data were non-destructive

Reporting for specific materials, systems and methods

We require information from authors about some types of materials, experimental systems and methods used in many studies. Here, indicate whether each material, system or method listed is relevant to your study. If you are not sure if a list item applies to your research, read the appropriate section before selecting a response.

Materials & experimental systems

n/a	Involved in the study
☒	☐ Antibodies
☒	☐ Eukaryotic cell lines
☒	☐ Palaeontology and archaeology
☒	☐ Animals and other organisms
☒	☐ Human research participants
☒	☐ Clinical data
☒	☐ Dual use research of concern

Methods

n/a	Involved in the study
☒	☐ ChIP-seq
☒	☐ Flow cytometry
☒	☐ MRI-based neuroimaging
